# A pancancer analysis of the carcinogenic role of receptor-interacting serine/threonine protein kinase-2 (RIPK2) in human tumours

**DOI:** 10.1186/s12920-022-01239-3

**Published:** 2022-04-26

**Authors:** Hanqun Zhang, Yan Ma, Qiuning Zhang, Ruifeng Liu, Hongtao Luo, Xiaohu Wang

**Affiliations:** 1 The First School of Clinical Medicine, Lanzhou University, Lanzhou, 730000 People’s Republic of China; 2 Institute of Modern Physics, Chinese Academy of Sciences, Lanzhou, 730000 People’s Republic of China; 3 University of Chinese Academy of Sciences, Beijing, 100049 People’s Republic of China; 4 Department of Oncology, Guizhou Provincial People’s Hospital, Guizhou, 550002 People’s Republic of China; 5 Lanzhou Heavy Ion Hospital, Lanzhou, 730000 People’s Republic of China

**Keywords:** RIPK2, Carcinogenesis, Human tumours, Mechanism, Pancancer

## Abstract

**Background:**

To explore the expression and carcinogenic mechanism of RIPK2 in human tumours, and to provide the theoretical basis for the further study of RIPK2.

**Methods:**

We used the TCGA, CPTAC, HPA databases to analyse the expression, mutation, and prognosis of RIPK2 in human tumours. Through the Cbioportal, Ualcan, TIMER2.0, and STRING websites, We understand the genetic variation, immune infiltration and enrichment analysis of RIPK2 related genes.

**Results:**

RIPK2 was highly expressed in most tumours (such as BRCA, COAD and LUSC, etc.), and the high expression of RIPK2 was correlated with tumour stage and prognosis. In addition, Amplification was the main type of RIPK2 in tumour mutation state, and the amplification rate was about 8.5%. In addition, RIPK2 was positively associated with tumour-infiltrating immune cells (such as CD8+ T, Tregs, and cancer-associated fibroblasts). According to the KEGG analysis, RIPK2 may play a role in tumour mainly through NOD-like signaling pathway and NF-kappaB signaling pathway. GO enrichment analysis showed that the RIPK2 is mainly related to I-kappaB kinase/NF-kappaB signaling, Ribonucleoprotein granule and Ubiquitin-like protein ligase binding.

**Conclusion:**

RIPK2 plays an important role in the occurrence, development and prognosis of malignant tumours. Our pancancer study provided a relatively comprehensive description of the carcinogenic effects of RIPK2 in different tumours, and provided useful information for further study of RIPK2.

**Supplementary Information:**

The online version contains supplementary material available at 10.1186/s12920-022-01239-3.

## Background

Cancer is a disease that threatens human health. Regardless of the level of a country’s development, cancer is a major cause of high morbidity and mortality in every country and region of the world. In recent decades, the incidence and mortality of cancer have increased significantly [1]. According to statistics, approximately 19.3 million new cancer cases were reported globally in 2020, and approximately 10 million people died from this disease [2]. In addition, with the growth of the population, ageing trends and poor lifestyles, the new incidence and mortality of cancer may continue to increase [3]. Therefore, the global control of cancer is necessary and urgent. Because, the occurrence and development of cancer are complex, it is imperative to understand the mechanism of its occurrence and development as well as clinical prognosis. Therefore, it is important to conduct pancancer expression analysis of all genes of interest or importance and evaluate their correlation with the molecular mechanism of cancer development and clinical prognosis. With the advent of new detection tools and detection equipment that have led to the rapid development of proteomics, transcriptomics and genomics, as well as the establishment of corresponding databases, it has become more convenient to obtain data, allowing the pancancer analyses to be performed.

Receptor interacting protein kinase 2 (RIPK2), also known as receptor-interacting serine/threonine kinase (RICK) or card-containing IL-1β converting enzyme associated kinase,CARDIAK), consists of an N-terminal serine/threonine kinase domain and a C-terminal CARD domain [4]. RIPK2 not only plays an important role in inflammatory and immune diseases [5], but also participates in tumour invasion and metastasis [6, 7]. In addition, RIPK2 also plays an important role in the tumour microenvironment [8]. However, a comprehensive pancancer analysis between RIPK2 and various tumour types has not been reported. Therefore, we used the TCGA, CPTAC, HPA, and GEPIA2 databases or websites to conduct pancancer analysis of RIPK2, and explore the molecular mechanism and clinical prognosis of RIPK2 in cancer.

## Materials and methods

### Gene expression analysis

We entered RIPK2 into the “Gene_DE” module of the TIMER2.0 website (http://timer.cistrome.org/), and obtained the difference in RIPK2 expression in different tumours and normal tissues in the TCGA database. There was no matched normal tissue in the TCGA database. Tumour tissue was obtained from the GEPIA2 (http://gepia2.cancer-pku.cn/) database and normal tissue was obtained from the GTEx database and the expression difference between the two groups was obtained. The cut-off value of P was less than 0.01, and the cut-off value of LogFC (fold change) was equal to 1. In the matched normal data column, “Select Match TCGA normal and GTEx Data” was selected. Using UALCAN tools (http://ualcan.path.uab.edu/index.html), and TCGA data analysis, we obtained RIPK2 expression in different tumour stages. In UALCAN total protein expression analysis (http://ualcan.path.uab.edu/home), we selected the "CPTAC analysis" module, and we input "RIPK2"to obtain the total protein expression in tumour tissue and normal tissue. Because "CPTAC" only had 6 total protein expression data from six tumours, we searched in the Oncomine database (http://www.oncomine.org/resource/login.html) to obtain total protein expression data from other tumours.

### Protein expression analysis

In the Human Protein Atlas (HPA) (https://www.proteinatlas.org/), we entered RIPK2 in the “Search” module on the home page,selected the “Search” button, and then selected the "Tissue" and "Pathology" columns. We obtained RIPK2 in human tumour tissue samples and corresponding normal tissue samples, and we then evaluated the protein expression of RIPK2 in tumour and normal tissues. The staining intensity was divided into four categories as follows: strong, medium, weak and negative. The protein expression score was determined by immunohistochemical (IHC) staining intensity and staining cell proportion with the following formula: negative–not detected; weak < 25%–not detected; weak combined with either 25–75% or 75%–low; moderate < 25%–low; moderate combined with either 25–75% or 75%–medium; strong < 25%–medium, strong combined with either 25–75% or 75%–high [9].

### Survival prognosis analysis

In the list of "Survival Anaylsis" in GEPIA2, we obtained the overall survival (OS) and disease-free survival (DFS) data of RIPK2 in a variety of human malignancies. The cut-off value was 50%. The Cox PH model was used to calculate the risk ratio, and the 95% confidence interval was described by a dashed line. The log-rank test was used in the hypothesis test, and overall survival and disease-free survival graphs were obtained from the “Survival Analysis” module of GEPIA2.

### Genetic variation analysis

In the cBioPortal (https://www.cbioportal.org/) dataset, we selected "TCGA extensive cancer atlas research", and entered "RIPK2" genetic variation characteristics, In the "Summary of Cancer Types", all results included change types and mutation results. and copy number changes in TCGA tumours were reviewed. We also used the "Comparison/Survival" module to obtain the total survival, disease-free, progression-free survival and disease-free survival data of cancer cases with RIPK2 mutations in the TCGA database. Kaplan–Meier survival charts were generated by the log-rank test, and P < 0.05 was considered significant.

### Immune infiltration analysis

In TIMER2.0 ((http://timer.cistrome.org/) the "Immune" page "Gene Expression" template and "RIPK2" input were selected, and "CD8+ T", "T-cell regulatory(Tregs)" and "Cancer-associated Fibroblast" were selected in the "Immune Infiltrates" to obtain the results of the relationship between RIPK2 and tumour immune infiltration. The TIMER, CIBERSORT, CIBERSORT-ABS, QUANTISEQ, XCELL, MCPCOUNTER and EPIC algorithms were used to estimate immune infiltration. The P value and partial correlation value were obtained by the purity adjusted Spearman's rank correlation test. The data are presented in a scatter plot. In addition, TCGA pancancer data were downloaded through UCSC Xena, and Spearman correlation analysis was performed in R (3.6.3). The correlation between RIPK2 and granzyme B (GZMB), perforin 1 (PRF1), and natural killer cell granule protein 7 (NKG7) was analysed by ggplot2 visualization.

### Enrichment analysis

First, the STRING tool (https://string-db.org/) was used to screen 50 experimentally verified RIPK2 binding proteins. On the STRING page, "Protein Name" was selected, "RIPK2" was the input and "Homo sapiens" was selected, and the following parameters were set: Full network; Evidence; Experiments; Low confidence (0.150), and no more than 50 Interactors, in 1st shell. After setting the parameters, the instructions were followed to proceed to the next step to obtain the RIPK2-binding protein. GEPIA2 was used for similar gene detection, and the first 200 similar genes were obtained. In addition, GEPIA2 was used to perform correlation analysis between the top five similar genes and RIPK2, Pearson correlation analysis was used for paired genes, and log2 TPM was applied to the dot plot to obtain the p value and correlation coefficient. RIPK2 was input into the Gene_Cor module on the TIMER website, and DCAF13, GDI2, NBN, NUDCD1 and OSGIN2 were entered into Gene Expression. After running the program, we obtained the heatmap. The data included the p value and partial correlation value obtained by purity-adjusted Spearman's rank correlation test. KEGG pathway analysis was conducted, we uploaded the data to DAVID (a database for annotation, visualization, and discovery coordination), and then selected the "OFFICIAL_GENE_SYMBOL" and "Homo sapiens" settings to obtain the functional annotation graph data. Enrichment pathway analysis was performed using a visual "Tidyr" (https://cran.r-project.org/web/packages/tidyr/index.html) and "ggplot2" ((https://cran.r-project.org/web/packages/ggplot2/index.html) in the R language package (version 3.6.2). In addition, we used the "clusterProfiler" (http://www.bioconductor.org/packages/release/bioc/html/clusterProfiler.html) in the R language package for GO enrichment analysis. P < 0.05 was considered statistically significant.

## Results

### Gene expression results of RIPK2

**Fig. 1:**
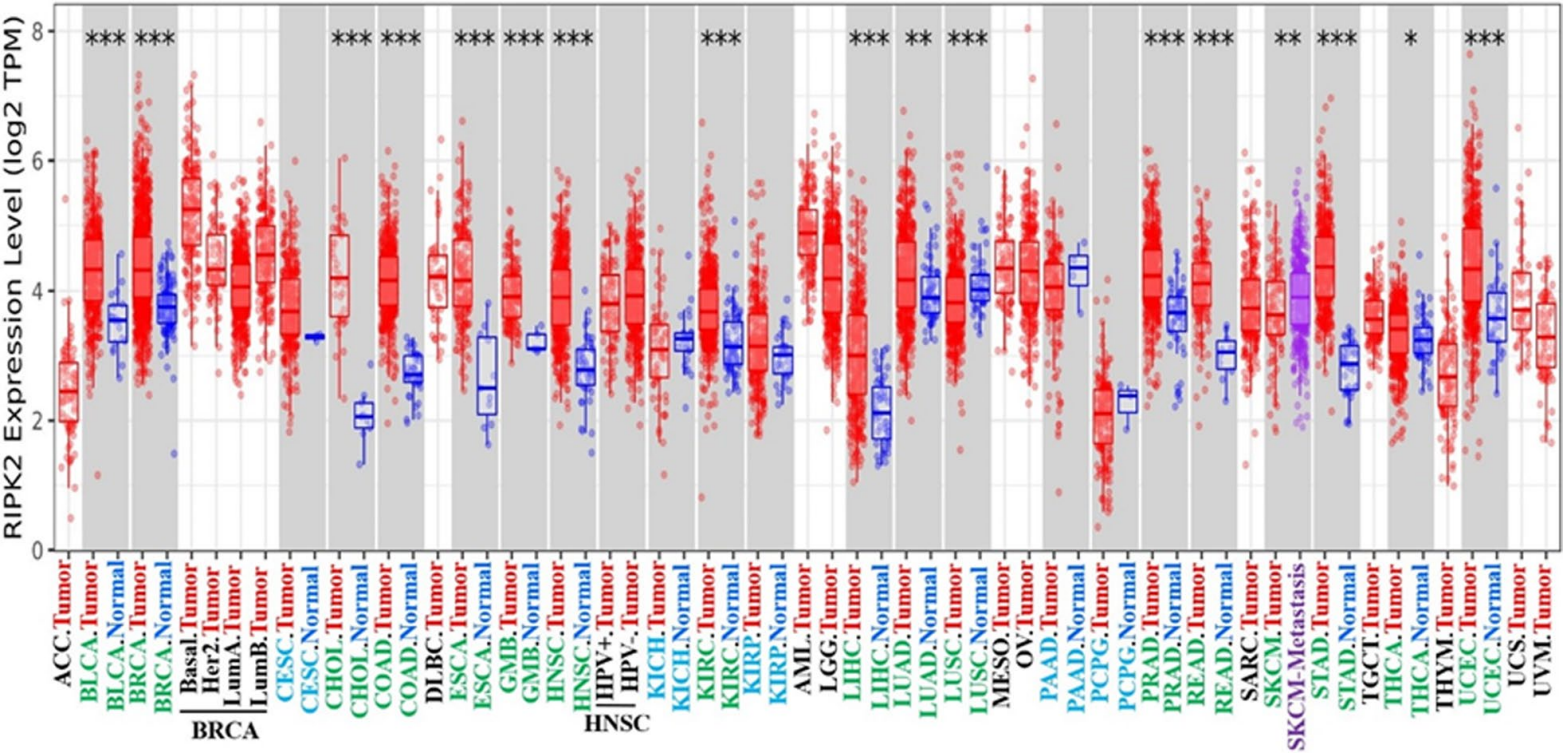
The expression status of the RIPK2 gene in different cancers or specific cancer subtypes was analyzed through TIMER2. The figure showed that RIPK2 expression in most malignant tumour tissues was higher than that in normal tissues, and it was statistically significant. (*P < 0.05; **P < 0.01; ***P < 0.001)

We obtained the expression difference of RIPK2 in various cancer types in the TCGA database through the TIMER2.0 website (Fig. 1). RIPK2 expression was found in Bladder urothelial carcinoma (BLCA), Breast invasive carcinoma (BRCA), Cholangio carcinoma (CHOL), Colon adenocarcinoma (COAD), Oesophageal carcinoma (ESCA), Glioblastoma multiforme (GMB), Head and Neck squamous cell carcinoma (HNSC), Kidney renal clear cell carcinoma (KIRC), Liver hepatocellular carcinoma (LIHC), Lung squamous cell carcinoma (LUSC), Prostate adenocarcinoma (PRAD), Rectum adenocarcinoma (READ), Stomach adenocarcinoma (STAD), Uterine Corpus Endometrial Carcinoma (UCEC) (P < 0.001), Skin cutaneous melanoma (SKCM), and Lung adenocarcinoma (LUAD) (P < 0.01) and Thyroid carcinoma (THCA) (P < 0.05). The expression in tumour tissues was higher than that in normal tissues.RIPK2 expression showed no difference between Cervical squamous cell carcinoma and endocervical adenocarcinoma (CESC), Kidney Chromophobe (KICH), Kidney renal papillary cell carcinoma (KIRP), Pancreatic adenocarcinoma (PAAD) and Pheochromocytoma and Paraganglioma (PCPG) tumour tissues and normal tissues (P > 0.05). In addition, in the TCGA, Adrenocortical carcinoma (ACC), Lymphoid Neoplasm Diffuse Large B-cell Lymphoma (DLBC), Acute Myeloid Leukemia (AML), Brain Lower Grade Glioma (LGG), Mesothelioma (MESO), Ovarian serous cystadenocarcinoma (OV), Sarcoma (SARC), Testicular Germ Cell Tumours (TGCT), Thymoma (THYM), Uterine Carcinosarcoma (UCS) and Uveal Melanoma (UVM) did not have matched normal tissue. Therefore, we searched for matched normal tissues in the GTEx database as a control for tumour tissues in the TCGA. We analysed these tumours and found that RIPK2 expression was higher in DLBC, LGG and OV than in normal tissues (P < 0.05) (Additional file 1: Fig. S1-A). No significant differences were observed in ACC, AML, SARC, TGCT, THYM and UCS tumours (P > 0.05) (Additional file 1: Fig. S1-B). Unfortunately, no matched normal tissue were available for MESO and UVM.

We next evaluated the total protein expression of RIPK2 in tumour tissues and normal tissues in BRCA, LUAD, COAD, UCEC, OV and KIRC in the CPTAC database. Unfortunately, the total protein expression of other cancers was not found in CPTAC. The total proteins expression in COAD, KIRC and UCEC tumour tissues was significantly higher than those in normal tissues (P < 0.05) (Additional file 1: Fig. S1-C), but no significant difference was observed in BRCA, LUAD and OV. Because the CPTAC database only had the total protein expression data of six tumours, we obtained the expression of SARC, HNSC and ESCA from the Oncomine database, and the differences were statistically significant (P < 0.05) (Additional file 1: Fig. S1-D).

We then analysed the correlation between the expression of RIPK2 in different stages and normal tissues. The expression of RIPK2 in BRCA, CESC, COAD, ESCA, HNSC, KIRC, READ, STAD and UCEC was significantly higher than that in normal tissues (P < 0.05) (Additional file 1: Fig. S1-E). Comparison of the results of BLCA (normal vs. stages 2, 3 and 4), CHOL (normal vs. stages 1, 2 and 4), KICH (normal vs. stage 4), KIRP (normal vs. stages 1, 2, 3 and 4), LIHC (normal vs. stages 1, 2 and 3), LUAD (normal vs. stages 1, 2 and 3), LUSC (normal vs. stage 1) and THCA (normal vs. stages 2 and 4) indicated that RIPK2 expression was higher in some stages than in normal tissues (Additional file 1: Fig. S1-F). However, no significant difference was found in pancreatic cancer (PAAD) and SKCM (P > 0.05). Other tumours, such as ACC, DLBC, MESO, UVM, OV, TGCT and UCS, did not have matched normal tissue.

### Immunohistochemical(IHC) results of RIPK2

**Fig. 2:**
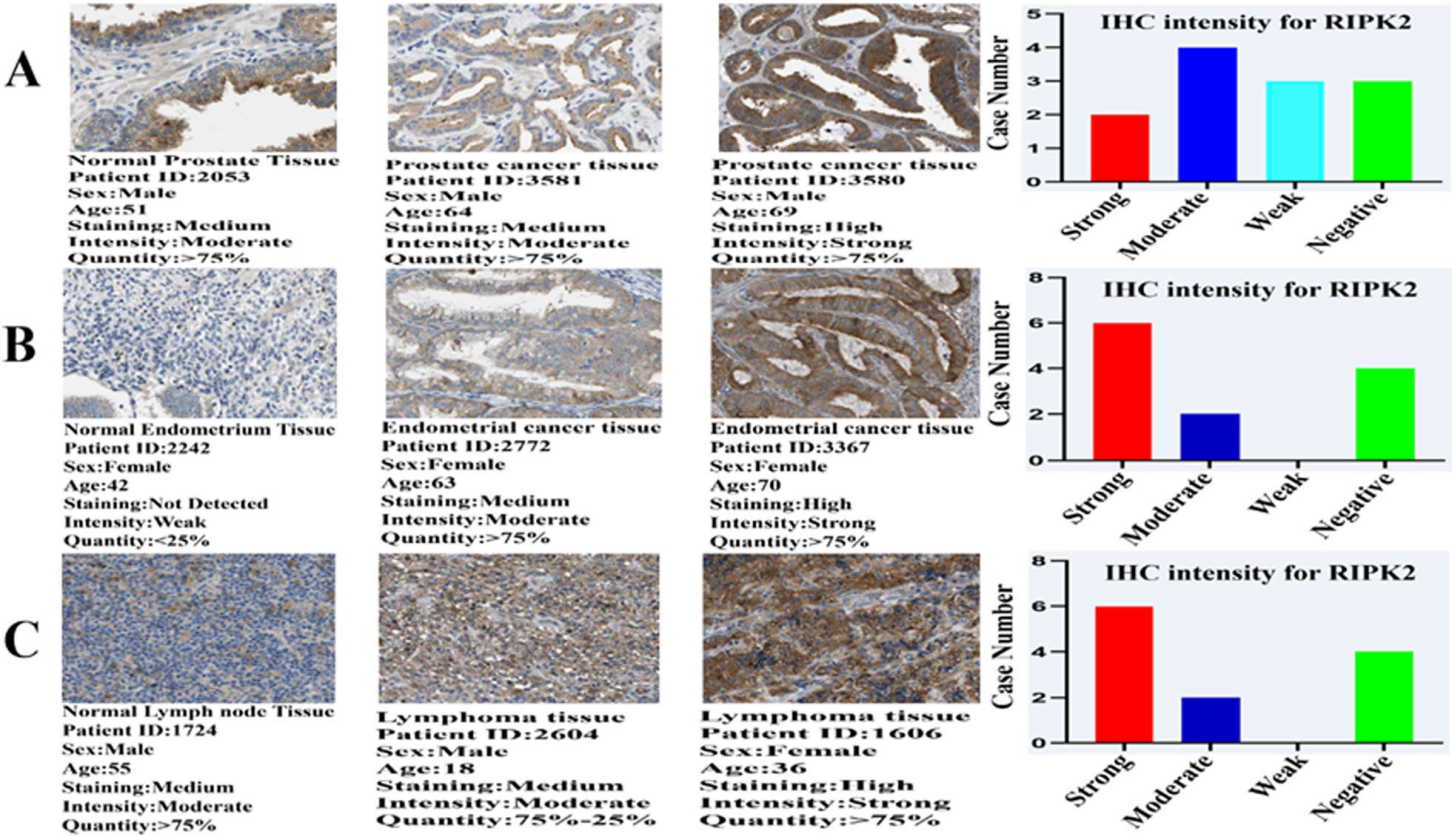
Immunohistochemical (IHC) images of normal and tumour tissues of RIPK2 from patients with Prostate cancer, Endometrial cancer, and Lymphoma (**A** Prostate cancer; **B** Endometrial cancer; **C** Lymphoma), and the intensity of the IHC of RIPK2. The bar graph shows the IHC intensity of RIPK2 (Prostate cancer:12 patients, Endometrial cancer:12 patients, and Lymphoma:11 patients). All IHC images and patient information were derived from the HPA

In HPA, we obtained immunohistochemical results of 19 various tumour tissues and corresponding normal tissues. The following basic information of patients was included: sex, age, ID number, antibody and subcellular localization. After analysing the immunohistochemical results, RIPK2 expression was only medium–high in Endometrial cancer (8/12 patients), Lymphoma (6/11 patients) and Prostate cancer (6/12 patients), and the proportion of medium–high expression of RIPK2 in endometrial cancer, lymphoma and prostate cancer tissues was higher than that of low or negative expression (Fig. 2). RIPK2 also showed medium–high expression in Liver cancer (4/12 patients), but it was still dominated by low expression and negative expression. RIPK2 was negatively expressed in Thyroid cancer (4/4 patients), but moderately expressed in normal tissues. In addition, RIPK2 was mainly negative in Lung cancer (9/11 patients), Glioma (9/12 patients), Testicular cancer (8/10 patients) and Renal cancer (9/12 patients). RIPK2 was mainly moderately expressed in other tumour tissues (Additional file 1: Fig. S2-A, S2-B, and S2-C).

### Survival analysis results of RIPK2

**Fig. 3:**
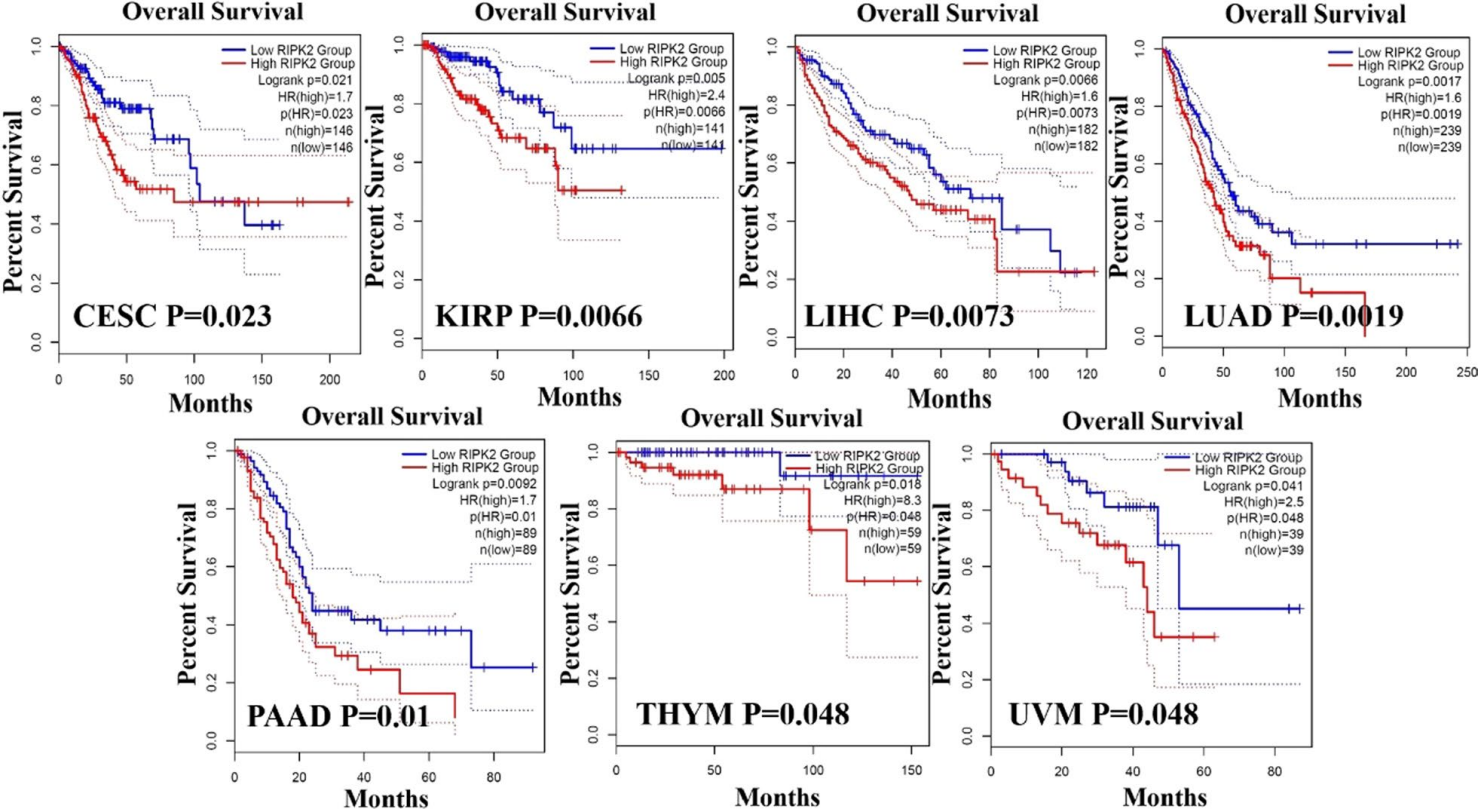
The overall survival of RIPK2 expression in CESC, KIRP, LIHC, LUAD, PAAD, THYM and UVM. We obtained a relationship between RIPK2 and survival prognosis of cancer from GEPIA2. Blue represents the Low RIPK2 Group; Red represents the High RIPK2 Group. Kaplan–Meier curves were all positive

We obtained survival and prognosis information on RIPK2 expression in various cancer types through GEPIA2. According to the expression level of RIPK2 in tumour tissues, RIPK2 was divided into high expression and low expression, and the correlation between different expression levels of RIPK2 and the prognosis of patients with different tumours was investigated. The overall survival of high RIPK2 expression in CESC, KIRP, LIHC, LUAD, PAAD, THYM and UVM was lower than that of low RIPK2 expression (P < 0.05) (Fig. 3). In disease-free survival, high RIPK2 expression was associated with KICH, KIRC, KIRP, LUSC, SARC, and UVM (P < 0.05) (Additional file 1: Fig. S3). No difference was found in other tumours.

### Genetic variation results of RIPK2

**Fig. 4:**
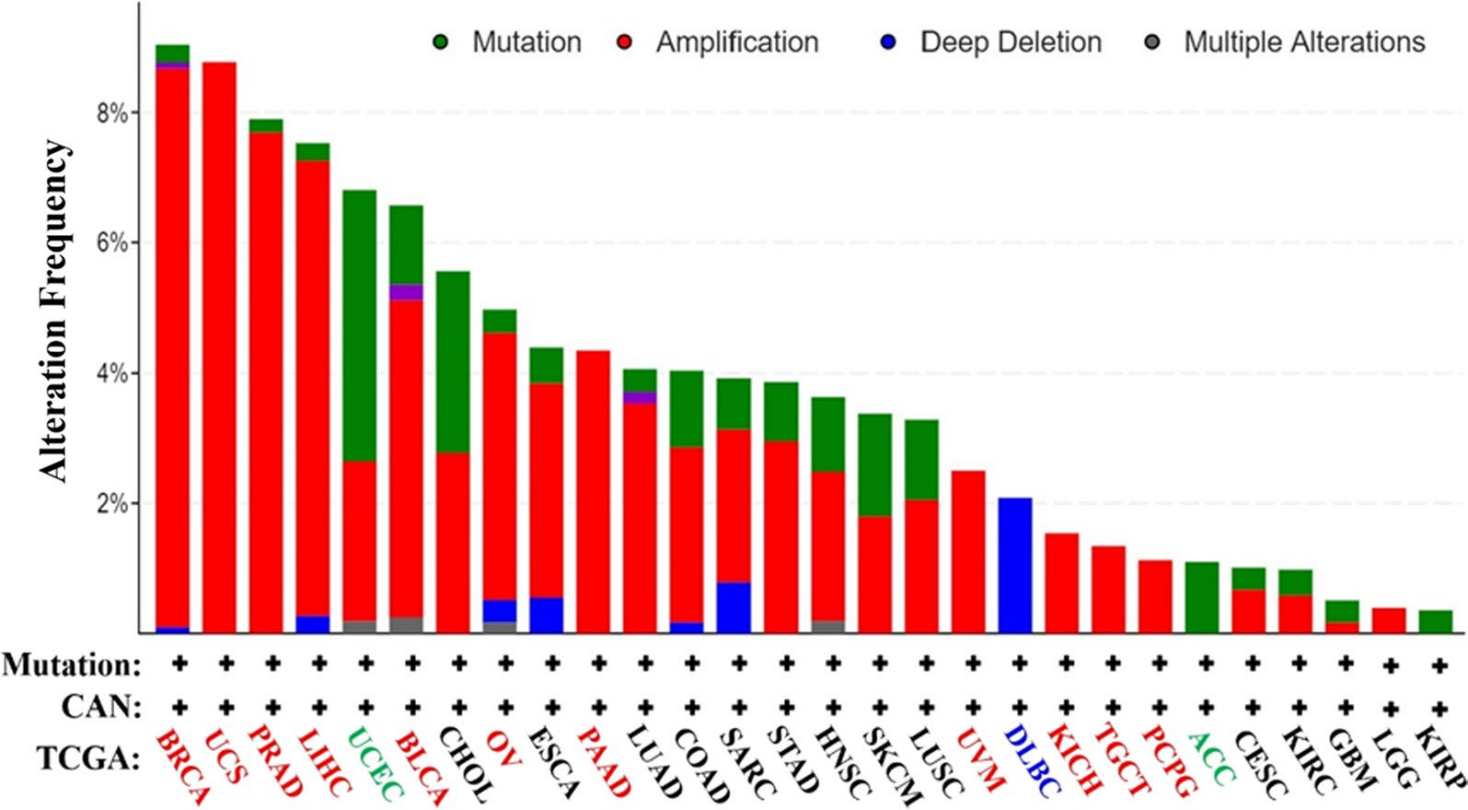
Mutation frequency of RIPK2 in malignant tumours. The figure showed that RIPK2 was the main type of Amplification in BRCA, UCS and PRAD, the main type of Mutation in UCEC, and the main type of Deep deletion in DLBC

**Fig. 5:**
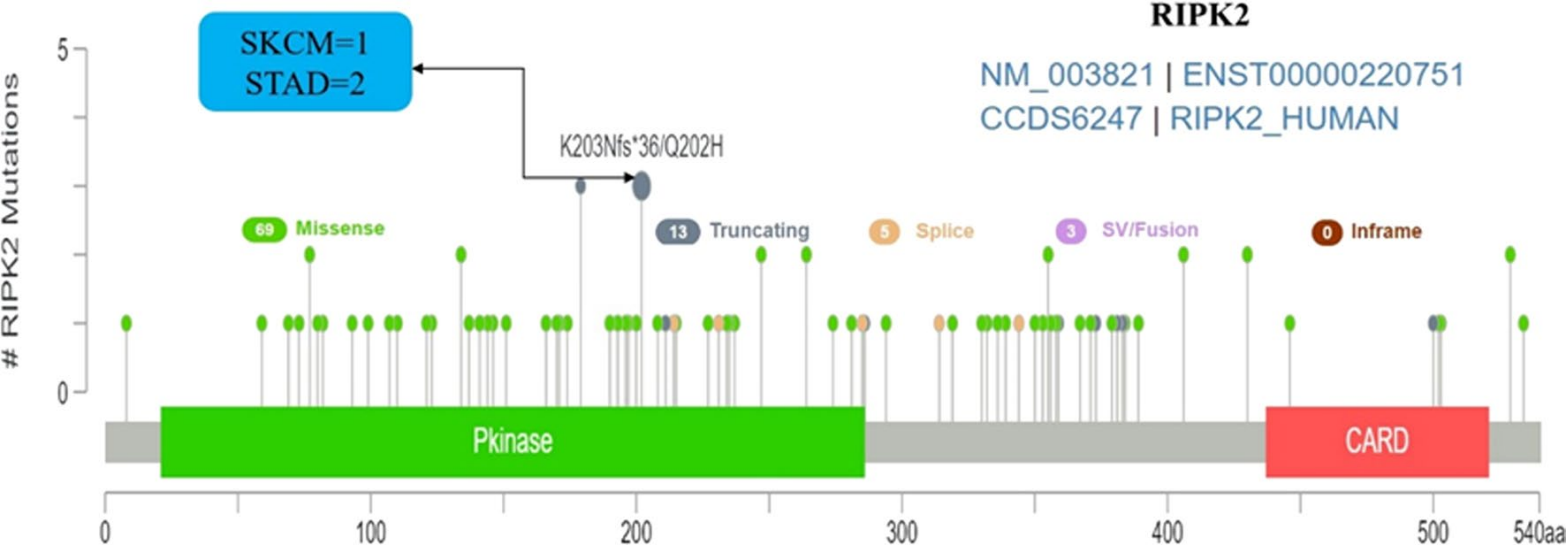
Mutation sites and number of cases of RIPK2 in malignant tumours. K203Nfs*36/Q202H was the site with the highest mutation frequency

From the TCGA database, we determined the expression status of genetic variation of RIPK2 in different tumours (Fig. 4). Among all the mutation expression states, the amplified type had the highest expression with an amplification rate of approximately 8.5%, mainly in BRCA and UCS. In UCEC, mutations were the main type of genetic variation with a mutation frequency of approximately 4%. In addition, a copy of the RIPK2 gene was missing in DLBC. We also evaluated the types, loci and number of cases of genetic variation of RIPK2. The main genetic variation types of RIPK2 were missense mutations and frame-shift mutations. Of note, in one case of SKCM and two cases of STAD, the protein was translated from Q (glutamine) to H (histidine) at site 202 in SKCM and from K (lysine) to N (asparagine) at site 203 in STAD (Fig. 5). We also explored the correlation between mutations in RIPK2 and clinical outcomes in patients with different tumours. Compared to patients without mutations, overall survival (P = 1.691e−4), progression-free survival (P = 0.0139), disease-free survival (P = 0.0227) and disease specificity (P = 2.379e−4) in OV were lower in the RIPK2 mutant group than in the nonmutant group (Additional file 1: Fig. S4-A). In addition, the RIPK2 mutant group was also lower than the nonmutant group in both PAAD and PRAD (Additional file 1: Fig. S4-B).

### Immune infiltration results of RIPK2

**Fig. 6:**
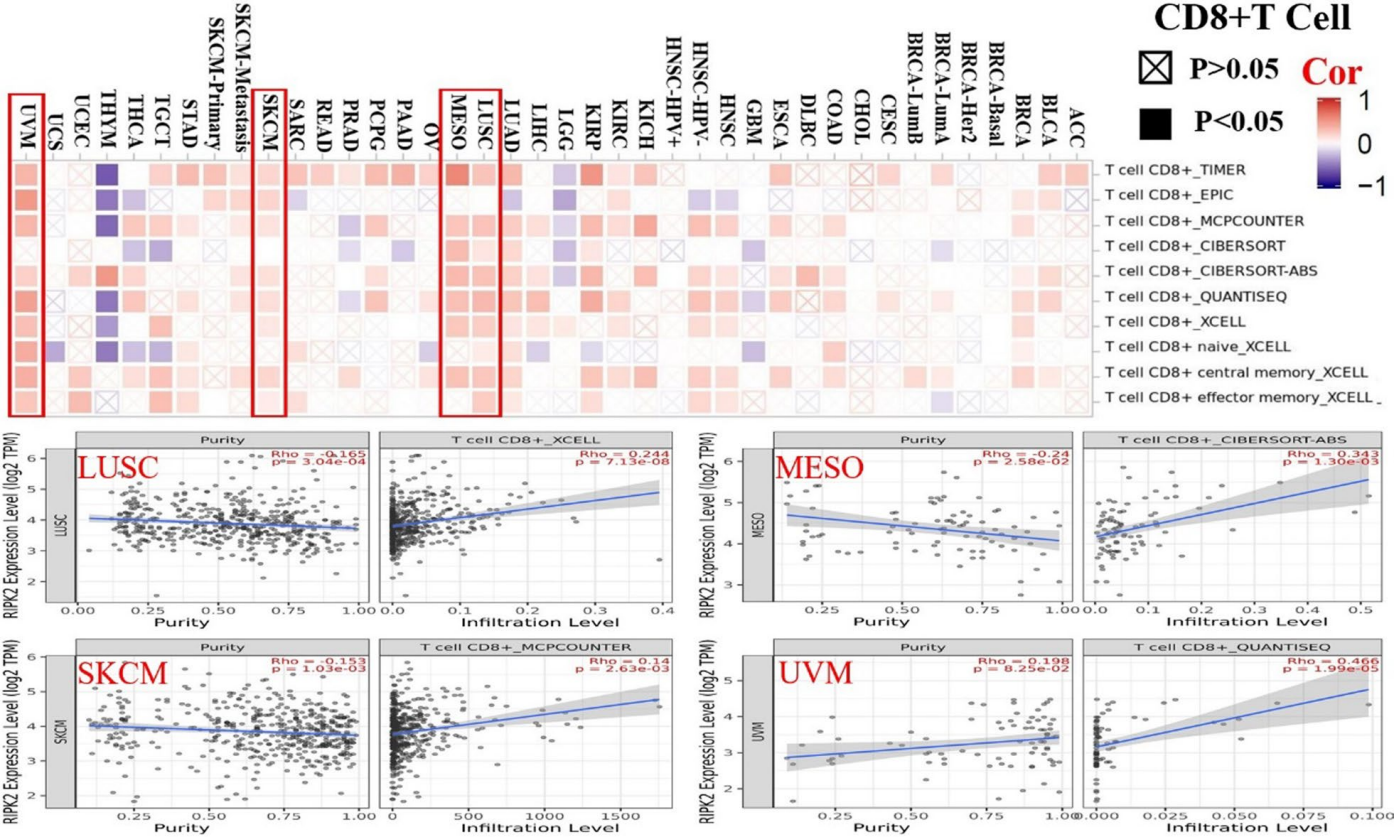
Correlation between RIPK2 expression and immune infiltration of CD8+ T cells. In LUSC, MESO, SKCM, and UVM, RIPK2 expression was positively correlated with CD8+ T cells expression. The TIMER, CIBERSORT, CIBERSORT-ABS, QUANTISEQ, XCELL, MCPCOUNTER and EPIC algorithms were used to estimate immune infiltration

To further understand the relationship between RIPK2 and tumour-infiltrating immune cells, we used the TIMER, CIBERSORT, CIBERSORT-ABS, QUANTISEQ, XCELL, MCPCOUNTER, and EPIC methods to investigate the potential relationship between the infiltration levels of different immune cells and RIPK2 gene expression in different cancer types in the TCGA. Based on our results, we found that the expression of RIPK2 and CD8+ T cells was positively correlated in LUSC, MESO, SKCM and UVM (Fig. 6). RIPK2 expression was positively correlated with Tregs in LIHC, LUSC, TGCT and THCA (Additional file 1: Fig. S5-A). In addition, we also found that RIPK2 was positively associated with cancer-associated fibroblasts (CAFs) in COAD, KIRP, and LUSC, but negatively associated with BRCA (Additional file 1: Fig. S5-B). To understand the relationship between RIPK2 expression and cytotoxic markers, TCGA pancancer data were downloaded through UCSC Xena, and we analysed the correlation of RIPK2 expression with GZMB, NGK7 and PRF1. We found that RIPK2 was positively correlated with GZMB, NGK7 and PRF1 in most cancers, however,RIPK2 was negatively correlated with PRAD and LGG, and it had no correlation with SKCM and DLBC (Additional file 1: Fig. S5-C).

### Enrichment analysis results of RIPK2

**Fig. 7:**
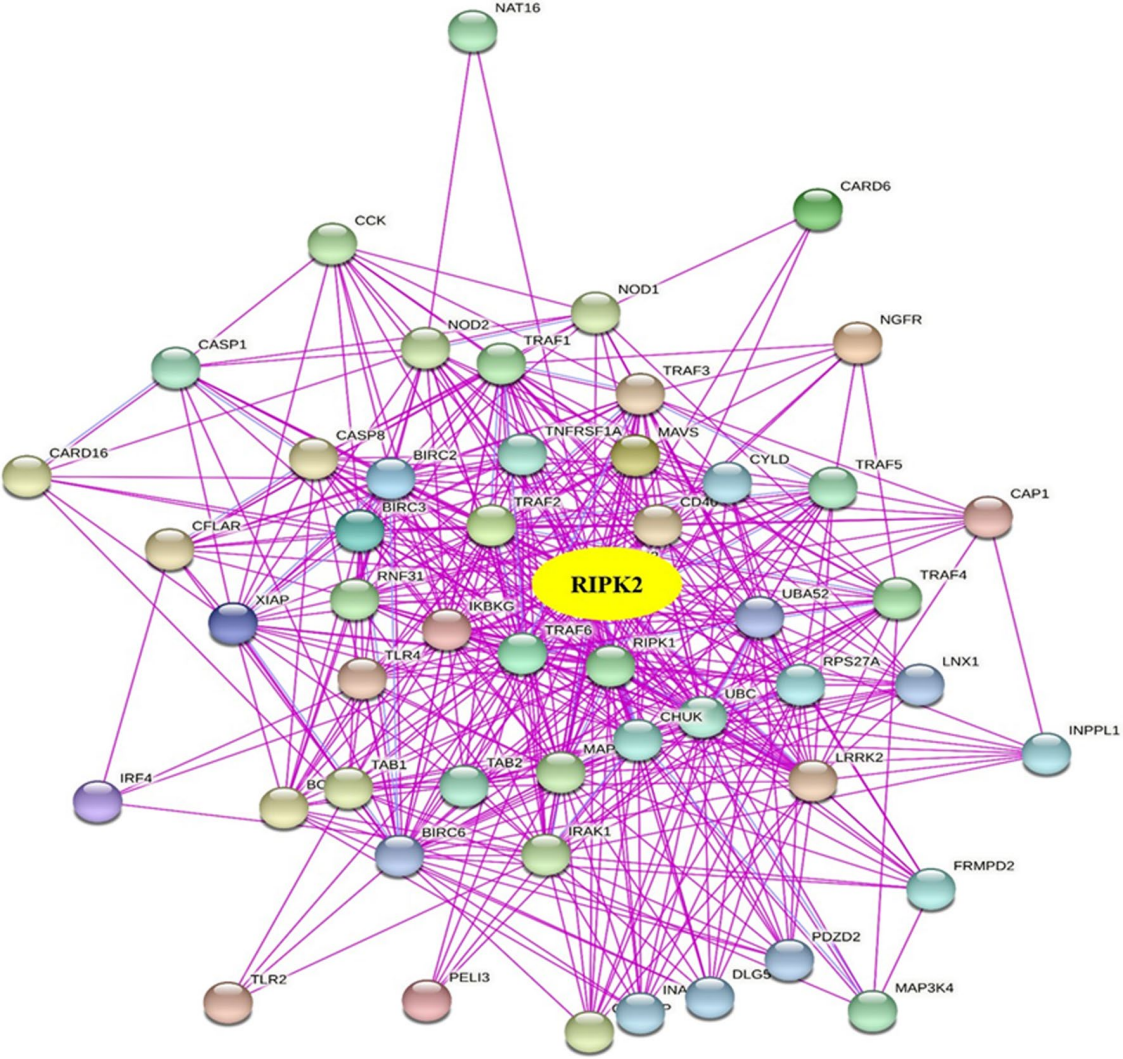
Fifty experiments confirmed the binding proteins and interaction networks interacting with RIPK2

To further understand the occurrence and development mechanism of RIPK2 in tumours, we screened RIPK2-binding proteins and genes related to RIPK2 expression. Using the STRING tool, we obtained 50 experimentally confirmed RIPK2-binding proteins and the interaction network between these proteins (Fig. 7). We used the GEPIA2 tool in combination with the TCGA and GTEx tumour expression data to obtain the top 200 genes associated with RIPK2 expression, We analysed the correlation between the expression of RIPK2 and the top five related genes, and the results showed that the expression of RIPK2 was positively correlated with these five genes (Additional file 1: Fig. S6-A).In addition, we used TIMER2.0 to obtain a heatmap of the comparison of RIPK2 with DCAF13, GDI2, NBN, NUDCD1 and OSGIN2, The corresponding heatmap results also showed that RIPK2 was positively correlated with the above five genes in tumours (Additional file 1: Fig. S6-B).

**Fig. 8:**
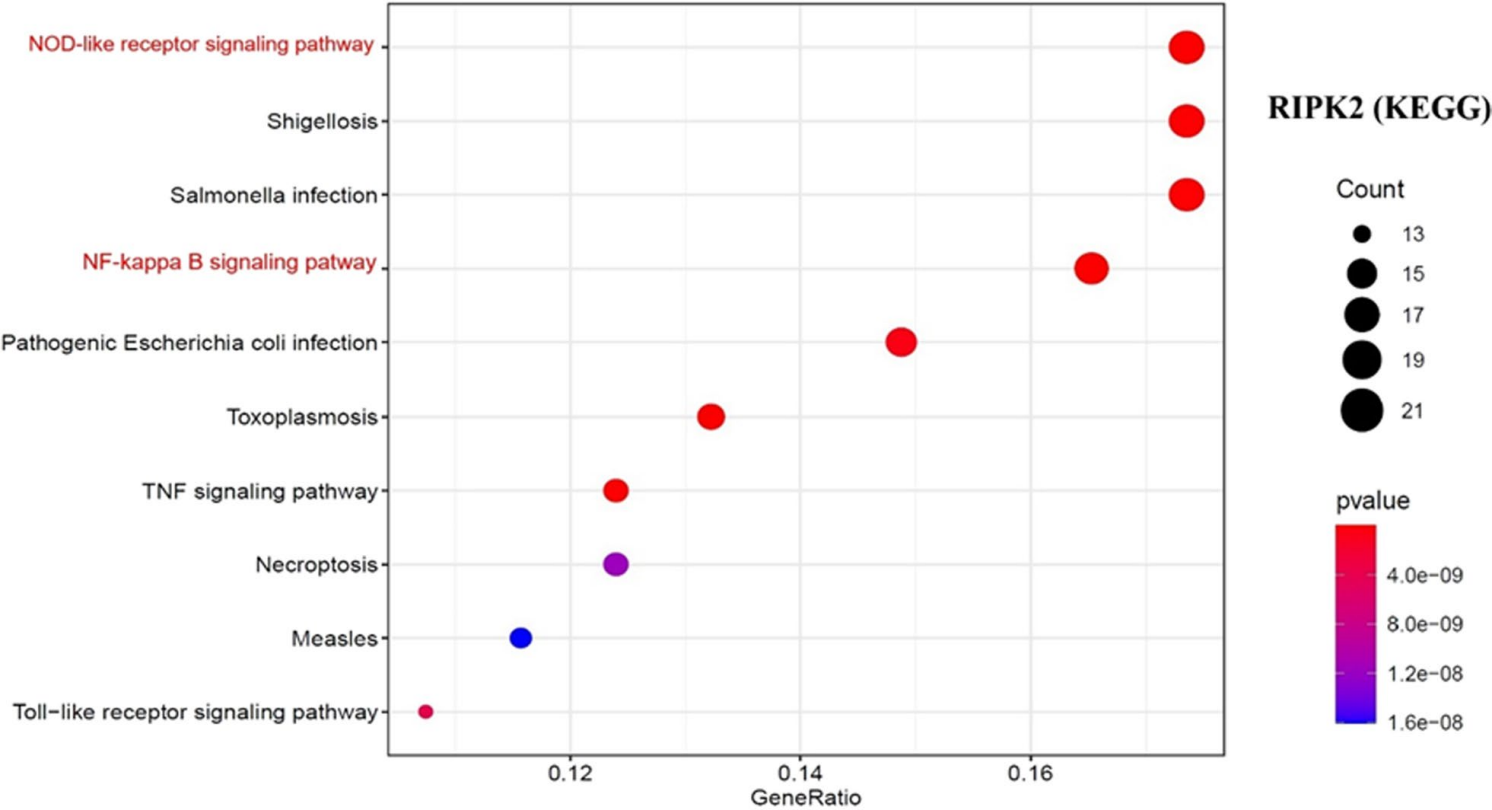
KEGG pathway analysis of RIPK2 interaction and expression related genes. The most common signaling pathways are NOD-like receptor signaling pathway and NK-kappa B signaling pathway

Finally, we combined two datasets of 50 RIPK2-interacting binding proteins with the 200 genes related to RIPK2 expression and performed KEGG and GO enrichment analyses. According to KEGG analysis, RIPK2 may play a role in tumours mainly through the NOD-like signalling pathway and NF-Kappa B signalling pathway (Fig. 8). The GO enrichment analysis results suggested that the mechanism of RIPK2 in tumours may involve I-kappaB kinase/NF-kappaB signalling, ribonucleoprotein granule and ubiquitin-like protein ligase binding (Additional file 1: Fig. S6-C, D and E).

## Discussion

Members of the receptor interacting protein kinase (RIPK) family are important sensors of intracellular and intracellular stress, and they play an important role not only in inflammation and the immune response, but also in the process of death induction [10]. RIPK contains seven members (RIPK1, RIPK2, RIPK3, RIPK4, RIPK5, RIPK6, and RIPK7), All RIPK members share a homologous kinase domain, but they have different functional domains. RIPK2 is characterized by an N-terminal serine/threonine kinase domain and a C-terminal CARD [4]. Activation of RIPK2 is mediated by binding to a polyubiquitin chain K63 (Lys63) at lysine 209 (K209) in its kinase domain, leading to oligomerization of RIPK2, which promotes the ubiquitination of the NEMO/IKKγ K63 complex and the recruitment of transforming growth factor beta-activated kinase 1 (TAK1) [11]. TAK1 activation leads to the activation of MAPK kinase signalling pathways (ERK, P38 and JNK) and NF-κB activation. In addition, TAK1 phosphorylation activates the IKK complex and ultimately leads to IκB dissociation [12], NF-κB activation and the release of proinflammatory cytokines (e.g., TNFα and IL-6) [13, 14]. RIPK2 also reduces the production of cytokines induced by IL-1, IL-18, and Toll receptors, which disrupts the proliferation triggered by T-cell receptors [15, 16]. In addition, RIPK2 is also associated with the antiapoptotic proteins Bcl-2, Bcl-XL, cIAP1 and Cflip to inhibit cell apoptosis [17–20]. In recent years, the relationship between RIPK2 and malignant tumours has attracted increasing attention, and there are an increasing number of reports on the relationship between RIPK2 and malignant tumours, especially the relationship between RIPK2 role in tumour occurrence and development [6–8]. However, it remains unclear whether a common molecular mechanism underlies the role of RIPK2 in the occurrence and development of different tumours. At present, no comprehensive pancancer analysis of RIPK2 has been reported. Therefore, we analysed RIPK2 in 33 different tumours through data from the TCGA, GTEx, HPA, CPTAC, GEPIA2 and other databases or websites. The molecular characteristics of gene expression, protein expression, gene mutation, clinical prognosis and immune infiltration were analysed and summarized to provide useful information for the further study of RIPK2.

According to RNA expression data, RIPK2 is highly expressed in most cancer tissues, and RIPK2 may play an oncogenic role in cancer. Among the total proteins, the expression of RIPK2 in COAD, KIRC, UCEC, SARC, HNSC and ESCA was higher than that in normal tissues. These results showed that total protein expression was consistent with RNA expression. In addition, the immunohistochemical results of RIPK2 were consistent with the results of RNA expression and total protein, but not consistent in Lung cancer and Thyroid cancer. which may be related to the small number of tissue specimens. Subsequently, we analysed the expression of RIPK2 in various pathological stages of cancer tissues, and found that the expression of RIPK2 in most pathological stages of cancer tissues was higher than that in normal tissues. However, the expression of RIPK2 did not increase with increasing pathological stages. This may be related to the insufficient number of clinical specimens, the small number of cases in each stage and the lack of statistical significance. Another possibility is related to the difference in the expression of the RIPK2 gene itself, which may be highly expressed in all malignant tumours. Such high expression directly affects the prognosis, but the mechanism or mode of action may not be clear. GEPIA2 database analysis revesled that high expression of RIPK2 was negatively correlated with low overall survival (OS) and disease-free survival(DFS). Many experimental and clinical studies have shown that cancer is a multifactor, multistage and multigene mutation accumulation process. In general, oncogenes are involved in the regulation of proliferation and differentiation. However, if the expression level of these genes is changed, this has the potential to induce tumour transformation [21]. In the clinical setting, the expression of RIPK2 in human Glioma tissues is higher than that in normal tissues, and this high expression level is positively correlated with tumour grade [22]. In colorectal cancer, the mRNA expression level of RIPK2 is related to some proteins involved in cancer occurrence, and patients with higher expression of RIPK2 also have higher expression of vascular endothelial growth factor (VEGF). Yan et al. [23] found that a high mRNA abundance of RIPK2 was significantly associated with poorer progression-free and metastasis-free survival in prostate cancer patients; these researchers also found that RIPK2 is frequently amplified and/or overexpressed in many other cancer types, and its mRNA overexpression was significantly associated with shortened overall survival in kidney papillary cell carcinoma, kidney clear cell carcinoma, kidney chromophobe, thyroid cancer, pancreatic adenocarcinoma, lung adenocarcinoma, uveal melanoma and thymoma. The findings of Yan et al. were consistent with our analysis results. Interestingly, Yan et al. also found that RIPK2 is required for prostate cancer metastasis but not for tumour growth, suggesting that RIPK2 plays an important role in prostate cancer metastasis. However, whether RIPK2 plays the same role in other cancers is unclear and needs to be further investigated. In addition, the expression of RIPK2 is positively correlated with IL-6, IL-8 and VEGF, which play a role in colorectal cancer, indicating that RIPK2 may be involved in the occurrence of colorectal cancer and that the expression level of RIPK2 is correlated with the prognosis of colorectal cancer [24]. In oral squamous cell carcinoma, the abnormal expression of RIPK2 can impair human immunity and interfere with cell dedifferentiation, apoptosis and proliferation, thus promoting carcinogenesis of the oral mucosa. However, with the development of oral squamous cell carcinoma, the expression level of RIPK2 gradually declines [25]. In addition, the prognosis of breast cancer is related not only to stage and treatment but also to molecular typing. In the molecular classification of breast cancer, triple-negative breast cancer (estrogen receptor [ER]/ progesterone receptor/human epidermal growth factor receptor-2 [HER2] negative) has a poorer prognosis than other types of breast cancer. The expression of RIPK2 in triple-negative breast cancer is higher than that of other molecular subtypes, and this high expression is related to the prognosis of triple-negative breast cancer [26], which may indicate that the abnormal expression of RIPK2 is one of the main factors leading to the poor prognosis of triple-negative breast cancer. In addition, high expression of RIPK2 leads to low relapse-free survival in triple-negative breast cancer patients [6]. These results indicate that RIPK2 plays an important role in the occurrence and development of cancer and that it is related to clinical prognosis, tumour grade and molecular typing. In addition,RIPK2 may be used as a new molecular marker and prognostic factor for cancer, especially for those tumour types with poor prognosis and special molecular types, indicating the need for further study.

The present study also investigated the expression status of genetic variation of RIPK2 in various tumours, including gene amplification, gene mutation and gene deletion. Among these, gene amplification was the most important expression type, and the amplification rate in BRCA and UCS was approximately 8.5%. ERBB2 is one of the amplification factors in BRCA, and the amplification of ERBB2 occurs in approximately 10–34% of BRCA; ERBB2 amplification is an important predictor of BRCA recurrence and survival [27, 28]. The gene amplification of ERBB2 is closely related to its protein overexpression [29]. RIPK2 is highly expressed and has a high amplification rate in BRCA. Therefore, through continuous basic research and clinical verification, RIPK2 is likely to become an important predictor of BRCA recurrence and survival in the future. In addition, gene amplification is related to drug resistance [30], and RIPK2 has been shown to be amplified in most cancers. Therefore, the amplification of RIPK2 is a new research direction in the field of cancer treatment resistance. In the TCGA, we found that RIPK2 mutation status was associated with overall survival, progression-free survival, disease-free survival, and disease specificity of OV, PAAD, and PRAD, while no association was found in LUAD, HNSC, and CESC. These results indicate that the occurrence, development and clinical prognosis of tumours are not determined by the mutations being spread across the protein, which indicates that there is a lack of recurrent hotspot mutations with the potential of a tumour suppressor pattern, suggesting that RIPK2 may act as a cancer suppressor gene. However, multiple clinical tissue, cell and animal studies have confirmed that RIPK2 may act as an oncogene [22, 23]. Furthermore, RIPK2 has not been reported as a tumour suppressor gene. It may act as a tumour suppressor gene in some cancers, but that role has not yet been identified. With the further study of RIPK2, there may be new findings or evidence that RIPK2 plays a role in cancer as a tumour suppressor gene.

The cancer-related immune environment is complex and mysterious, which is partly reflected in the diversity of the immune response and the spatial and temporal heterogeneity of the developing tumour [31], and the effective regulation of cancer immunity can prevent cancer metastasis and invasion. In some cancers, the presence of invasive CD8+ T lymphocytes is associated with improved prognosis, while CD4+ T lymphocytes, myeloid suppressor cells (MDSCs) and T regulatory cells (Tregs) are negatively correlated with survival [32]. Li et al. [33] used the TIMER algorithm to calculate the phenotype of each immune cell in the KIRC tumour microenvironment, including T cells (CD4+ T cells and CD8+ T cells), B cells, macrophages, neutrophils and dendritic cells. and they reported that with the exception of CD4+ T cells, most immune cell phenotypes show higher infiltration levels in specimens with high RIPK2 expression. This finding indicates that the high expression of RIPK2 is positively correlated with the expression of immune cells and is involved in the prognosis of cancer patients. Zhang et al. [8] used in situ and subcutaneous tumour models of RIPK2-deficient bladder cancer-bearing animals and observed an increase in tumour infiltration of CD11b + Gr1Himyeloid-derived suppressor cells (MDSCs) accompanied by a decrease in CD8+ T lymphocytes, CD4+ T lymphocytes, and NK cells, promoting tumour invasion and metastasis. Therefore, we used the QUANTISEQ, XCELL, MCPCOUNTER and EPIC methods to analyse the relationship between RIPK2 and CD8+ T, T regulatory cells and cancer-related fibroblasts. We found that RIPK2 was positively correlated with CD8+ T, T regulatory cells, and cancer-related fibroblasts in most cancers, whereas in BRCA, RIPK2 is negatively correlated with cancer-related fibroblasts. Studies have shown that cancer-associated fibroblasts in primary tumours enhance tumour cell invasion by secreting cancer-associated fibroblast chemokine (C-X-C motif) ligand 1 (CXCL1) and interacting with C-X-C motif chemokine receptor 2 (CXCR2) in tumour cells [34]. High expression of RIPK2 promotes the occurrence and development of tumours. Therefore, RIPK2 is negatively correlated with cancer-related fibroblasts in BRCA, which may inhibit the invasion and metastasis of BRCA, suggesting that RIPK2 plays a different role in malignant tumours. In conclusion, this is the first study to propose a relationship between RIPK2 and CD8+ T cells, Tregs and cancer-related fibroblasts in malignant tumours, indicating that RIPK2 and immune cells are jointly involved in the formation and development of cancer. However, the specific mechanism of action is still unclear and requires further study.

Cytotoxic markers play an important role in cancer detection, cancer treatment and cancer prognosis. Understanding the expression level of cytotoxic markers has a guiding role in cancer detection and efficacy evaluation. Granulation B is produced by cytotoxic T lymphocytes and natural killer cells, and it is a toxic granulation secretase with killing activity [35, 36]. It has been reported that low GZMB expression is associated with tumour metastasis [37].NKG7 is a complete membrane protein that is expressed in cytotoxic particles of lymphocytes [38], and it plays an important role in the development and metastasis of cancer [39]. PRF1 belongs to the membrane attack complex/PRF (MACPF) protein family, and it is mainly involved in the granular killing activity of cytotoxic T lymphocytes (CTLs) and NK cells [40]. In addition, the expression level of PRF1 is related to tumour prognosis [41]. It has been reported that GZMB and PRF1 are highly expressed in tumour tissues, and it was found that tumour tissues with high expression of GZMB and PRF1 have higher infiltration of CD8+ T cells and better tumour prognosis [42, 43]. The expression of NKG7 is highly correlated with cytotoxicity and CD8+ T cells, and NKG7 was upregulated in tumour-specific CD8+ T cells. However, decreased NKG7 expression alters the number, transport and calcium release of cytosolic particles, leading to reduced CD8+ T cell-mediated tumour cell killing [44, 45]. The present study found that RIPK2 was positively correlated with the expression of GZMB, NKG7 and PRF1 in cancer but negatively correlated with PRAD and LGG, and we found that RIPK2 had no correlation with SKCM and DLBC. However, high expression of RIPK2 led to poor prognosis, while high expression of GZMB, NKG7 and PRF1 led to better prognosis. Nonetheless,RIPK2 was only correlated with GZMB but not with NKG7 and PRF1. Moreover, RIPK2 was negatively correlated with CD8+ T cells, suggesting that RIPK2 may play a dominant role in THYM prognosis compared to GZMB, NKG7 and PRF1. In CESC, KIRP, LUAD and PAAD, the high expression of RIPK2 had a poor prognosis, but in CESC, KIRP, LUAD and PAAD, the expression of RIPK2 was positively correlated with that of GZMB, NKG7 and PRF1. These results indicated that the prognosis of CESC, KIRP, LUAD and PAAD is not determined by one or several genes but may be determined by other factors. At present, the relationship of RIPK2 with GZMB, NKG7 and PRF1 in cancer is still unclear, and this still needs to be explored in the occurrence, development, treatment and prognosis of cancer.

After obtaining the RIPK2-binding proteins from the STRING database and the genes related to RIPK2 expression from GEPIA2, we performed enrichment analyses. Our analyses revealed that the occurrence and development mechanisms of RIPK2 in human tumours were mainly associated with NOD-like signalling pathways, the NF-kappa B signalling pathway, I-kappa B kinase/NF-kappa B signalling, ribonucleoprotein granule and ubiquitin-like protein ligase binding. These findings provide useful information for the in-depth study of RIPK2 in human tumours and a theoretical basis for the development of drug targets. Recent studies have shown that RIPK2 has a variety of mechanisms of action in malignant tumours and can affect sensitivity to chemotherapy and targeted drugs. RIPK2 inhibits the proliferation and apoptosis of glioma cells by activating the NF-κB pathway and the mitogen-activated protein kinase (P38) pathway, and it inhibits tumour growth by regulating TRAF3 expression instead of the NF-κB pathway [22]. In RIPK2-expressing BRCA cells, the growth of BRCA cells is mainly inhibited by decreasing the expression of nuclear factor-kappa B (NF-kappa B) and C-Jun N-terminal kinase (JNK). In the BRCA mouse model, RIPK2 knockout increases the chemotherapy sensitivity of docetaxel, inhibits tumour volume and reduces lung metastasis [6]. In addition, RIPK2 promotes triple-negative breast cancer cell migration and invasion through activation of the NF-kappa B and JNK pathways [26]. In gastric cancer, downregulation of RIPK2 inhibits the growth, apoptosis and migration of gastric cancer cells by inhibiting the NF-κB signalling pathway. In sarcoma, gefitinib blocks the invasion and metastasis of tumour cells by inhibiting RIPK2 to reprogram macrophages [46]. In addition, RIPK2 also plays a role in hepatocellular carcinoma by affecting the expression of EMT-related genes [47]. The above studies indicate that the RIPK2 mechanism of action in malignant tumours is complex and diverse. There may be multiple mechanisms in one tumour, and multiple tumours may have a joint mechanism. The most common mechanism of RIPK2 in malignant tumours may occur through the NF-κB signalling pathway, and the NF-κB signalling pathway is closely related to tumour occurrence and development [8] as well as chemotherapy drug sensitivity [48], radiotherapy sensitivity [49], targeted therapy sensitivity [50] and immunotherapy [51]. Therefore, the RIPK2/NF-κB signalling pathway is an important pathway to improve the sensitivity of chemoradiotherapy, targeted therapy and immunotherapy for malignant tumours.

## Conclusions

To summarize, this is the first study to comprehensively analyse the correlation between RIPK2 expression and clinical prognosis, genetic variation, immune cell infiltration and the molecular mechanism of tumours. The present study improves our understanding of the role of RIPK2 in the occurrence and development of malignant tumours, and it provides useful information for further in-depth study of RIPK2 and a theoretical basis for drug development.

## Supplementary Information

**Additional file 1.** Supplementary figures.

## Data Availability

The TCGA data set, the HPA data and the Oncomine data were obtained from open data bases. The TCGA data set can be obtained from the following url: https://www.cancer.gov/about-nci/organization/ccg/research/structural-genomics/tcga.The HPA data obtained from the link below: https://www.proteinatlas.org/. The Oncomine data obtained from the link below: http://www.oncomine.org/resource/login.html.

## Abbreviations

ACC: Adrenocortical carcinoma
BLCA: Bladder urothelial carcinoma
BRCA: Breast invasive carcinoma
CESC: Cervical squamous cell carcinoma and endocervical adenocarcinoma
CHOL: Cholangio carcinoma
COAD: Colon adenocarcinoma
DLBC: Lymphoid Neoplasm Diffuse Large B-cell Lymphoma
ESCA: Esophageal carcinoma
GMB: Glioblastoma multiforme
HNSC: Head and Neck squamous cell carcinoma
KICH: Kidney Chromophobe
KIRC: Kidney renal clear cell carcinoma
KIRP: Kidney renal papillary cell carcinoma
LAML: Acute Myeloid Leukemia
LGG: Brain Lower Grade Glioma
LIHC: Liver hepatocellular carcinoma
LUAD: Lung adenocarcinoma
LUSC: Lung squamous cell carcinoma
MESO: Mesothelioma
OV: Ovarian serous cystadenocarcinoma
PAAD: Pancreatic adenocarcinoma
PCPG: Pheochromocytoma and Paraganglioma
PRAD: Prostate adenocarcinoma
READ: Rectum adenocarcinoma
SARC: Sarcoma
SKCM: Skin Cutaneous Melanoma
STAD: Stomach adenocarcinoma
TGCT: Testicular Germ Cell Tumours
THCA: Thyroid carcinoma
THYM: Thymoma
UCEC: Uterine Corpus Endometrial Carcinoma
UCS: Uterine Carcinosarcoma
UVM: Uveal Melanoma
