# Supplementary material for: A pancancer analysis of the carcinogenic role of receptor-interacting serine/threonine protein kinase-2 (RIPK2) in human tumours
Source: BMC Med Genomics. 2022 Apr 26;15:97. doi: 10.1186/s12920-022-01239-3 (PMC9040268; doi:10.1186/s12920-022-01239-3)

**A pancancer analysis of the carcinogenic role of Receptor-interacting serine/threonine protein kinase-2 (RIPK2) in human tumours**

**Supplementary Information**

**Figure S1-A**.The expression of RIPK2 in DLBC, LGG and OV. The expression of RIPK2 in DLBC, LGG and OV tumour tissues was higher than that in normal tissues, The differences were statistically significant.(*P<0 .05; **P<0 .01; ***P< 0.001). (GTEx dataset).


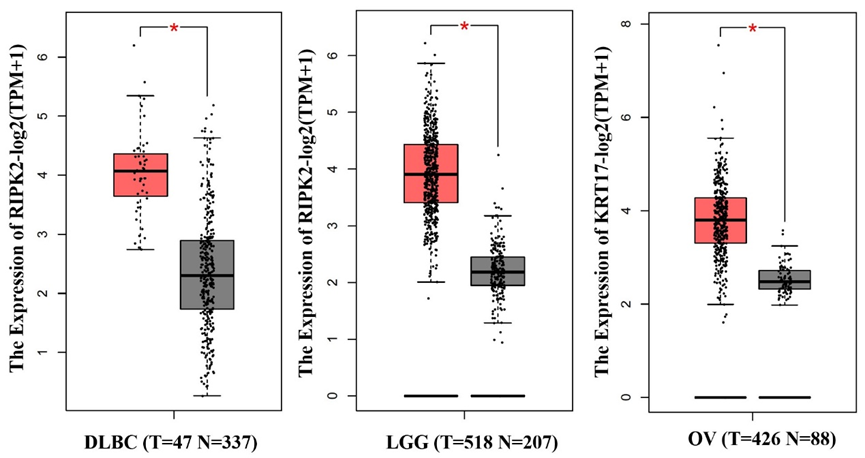


**Figure S1-B.** The expression of RIPK2 in ACC, AML, SARC, TGCT, THYN and UCS. No significant differences were observed in ACC, AML, SARC, TGCT, THYM and UCS tumours (P>0.05). (GTEx dataset).


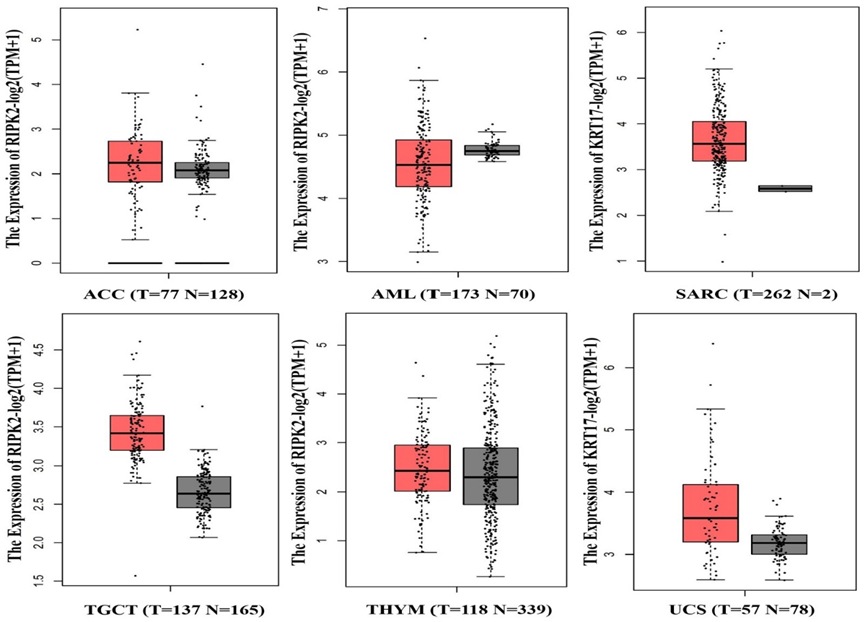


**Figure S1-C.** Total protein expression of RIPK2 in COAD, KIRC and UCEC. RIPK2 was highly expressed in these tumour tissues, and the differences were statistically significant.（*P<0 .05; **P<0 .01; ***P< 0.001）.（CPTAC dataset）.


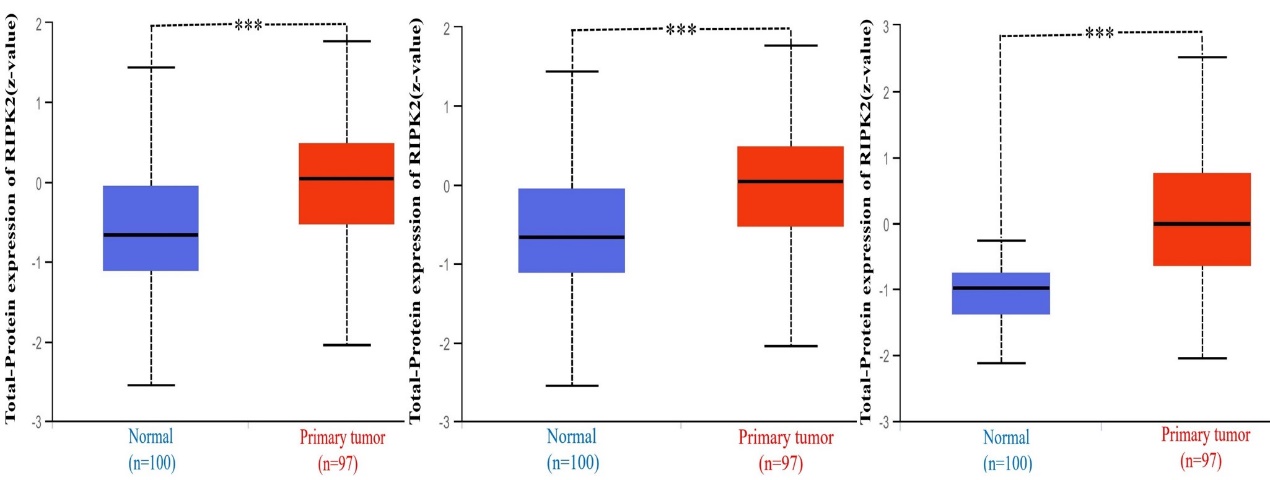


**Figure S1-D.** The expression of total proteins of RIPK2 in SARC, ESCA and HNSC. In addition, RIPK2 was highly expressed in these tumour tissues, and the differences were statistically significant. (Oncomine dataset).


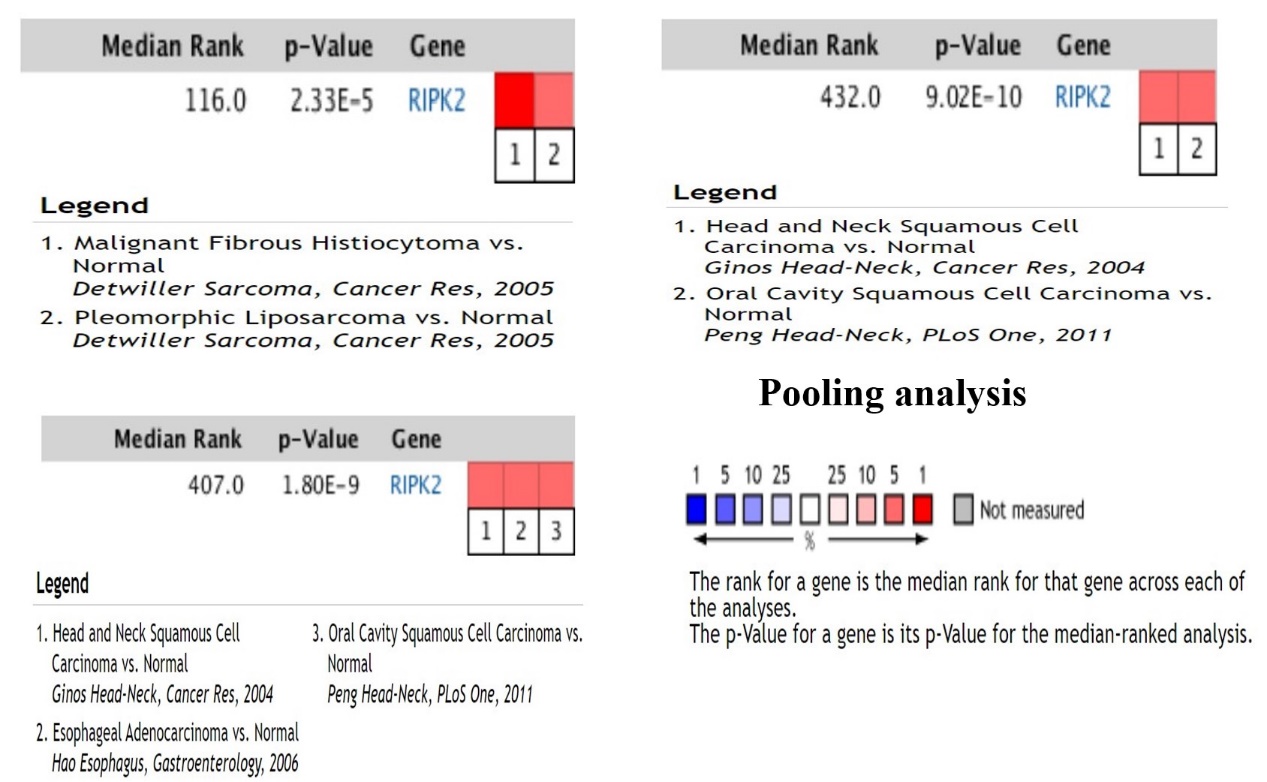


**Figure S1-E.** The expression of RIPK2 in different tumour pathological stages and normal tissues. In BRCA,CESC, COAD,ESCA,HNSC,KIRC,READ,STAD, and UCEC, RIPK2 expression in stage 1, 2, 3, and 4 was higher than that in normal tissues, and the differences were statistically significant.(*P<0 .05; **P<0 .01; ***P< 0.001). (TCGA dataset).


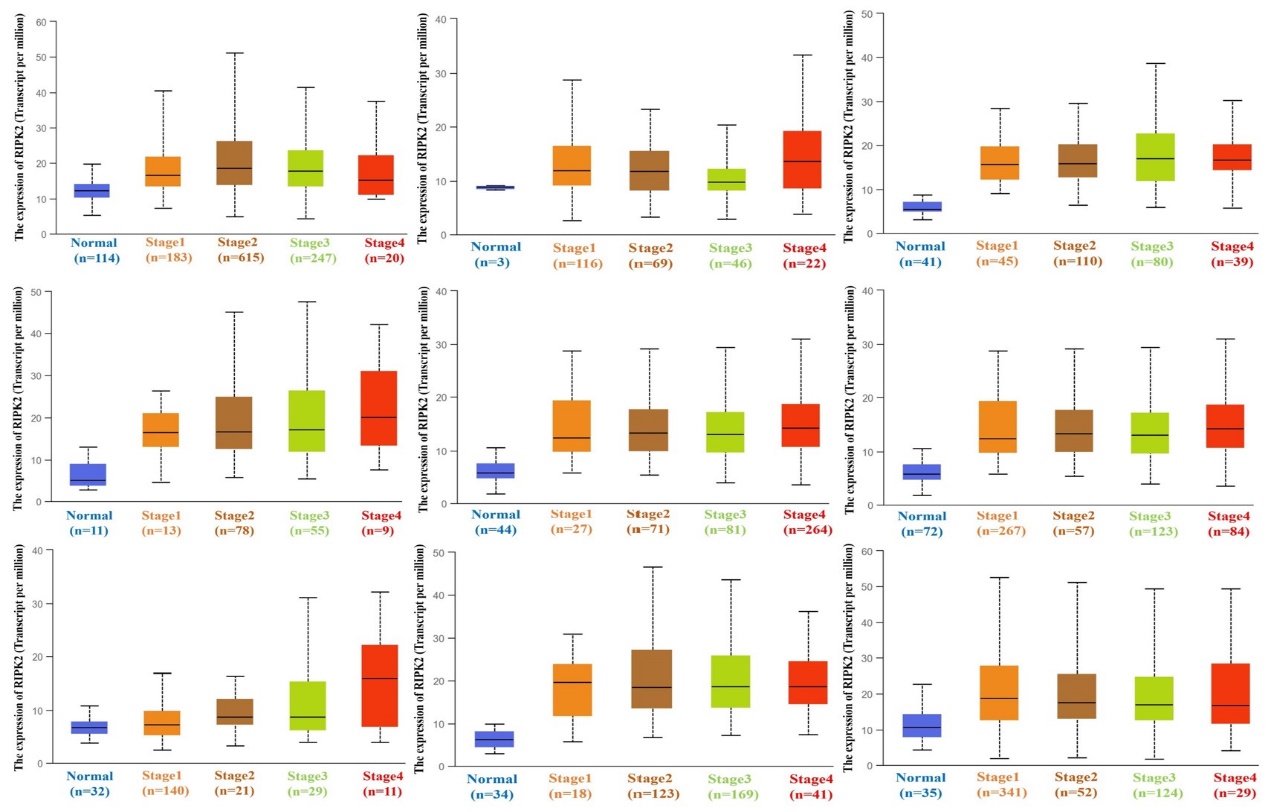


**Figure S1-F.** The expression of RIPK2 in BLCA, CHOL, KICH, KIRP, LIHC and LUAD in different tumour pathological stages and in normal tissues. In BLCA (stage 2,3,and 4), CHOL(stage1,2,and 4), KICH (stage4), KIRP(stage1,2,3, and 4), LIHC (stage1,2,and 3), LUAD(stage1,2,and 3) ,LUSC(stage 1),and THCA(stage 2,and 4), RIPK2 expression was higher than that in normal tissues, and the difference was statistically significant.(*P<0 .05; **P<0 .01; ***P< 0.001).(TCGA dataset).


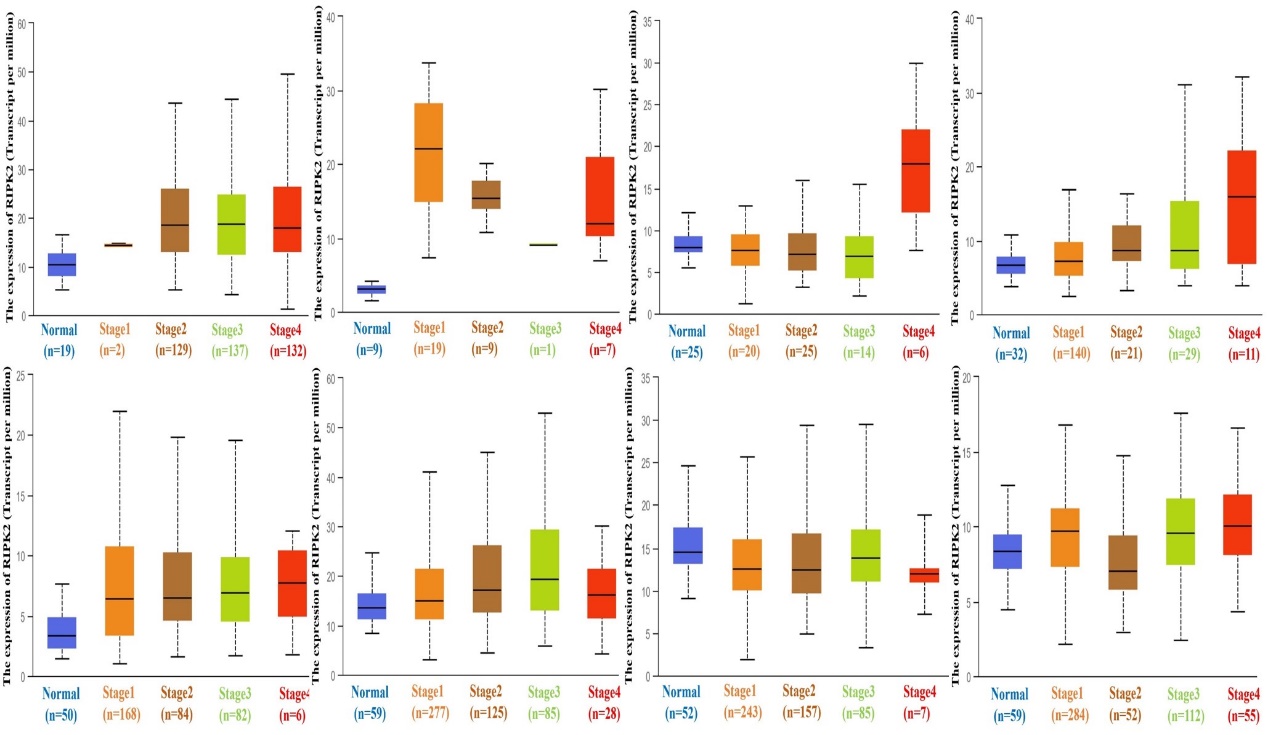


**Figure S2-A.** Immunohistochemical (IHC) images of normal and tumour tissues of RIPK2 from patients with Breast cancer, Glioma, Melanoma, Ovarian cancer,Pancreatic cancer and Skin cancer. All IHC images and patient information were derived from the HPA.


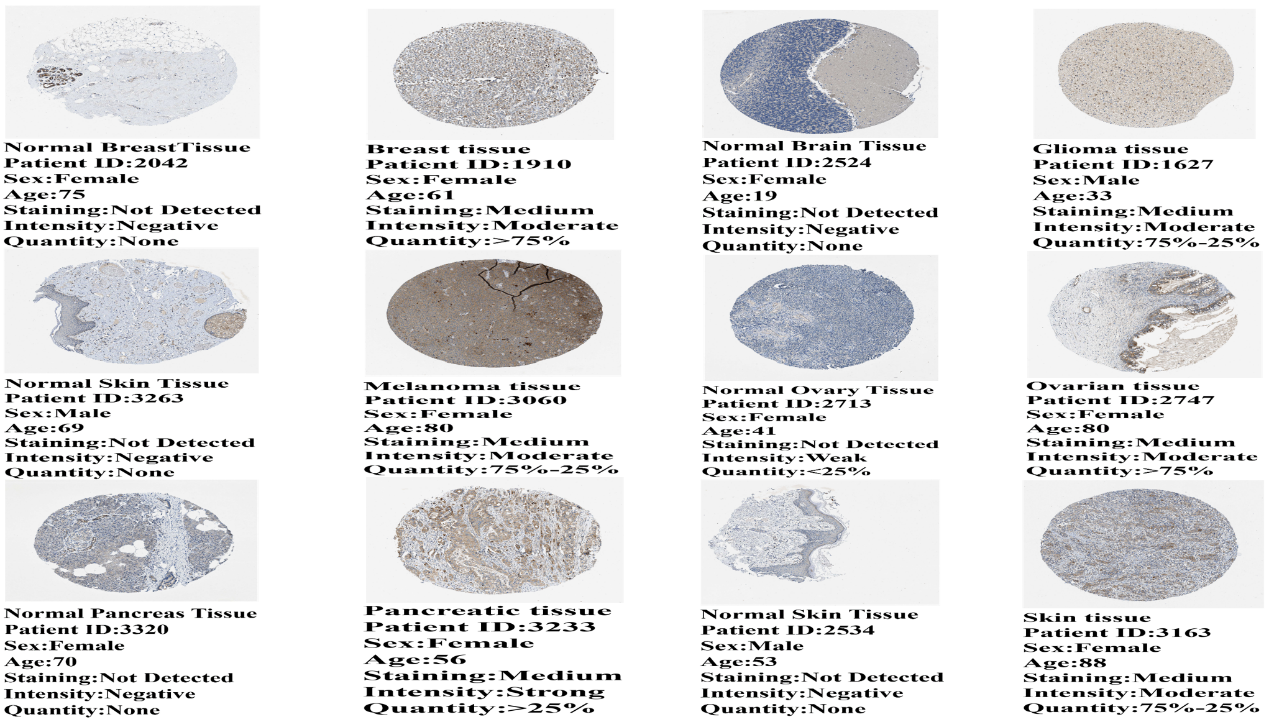


**Figure S2-B.** IHC images of normal and tumour tissues of RIPK2 from patients with Colorectal cancer, Renal cancer, Urothelial cancer, Cervical cancer, Testis cancer and Thyroid cancer. All IHC images and patient information were derived from the HPA.


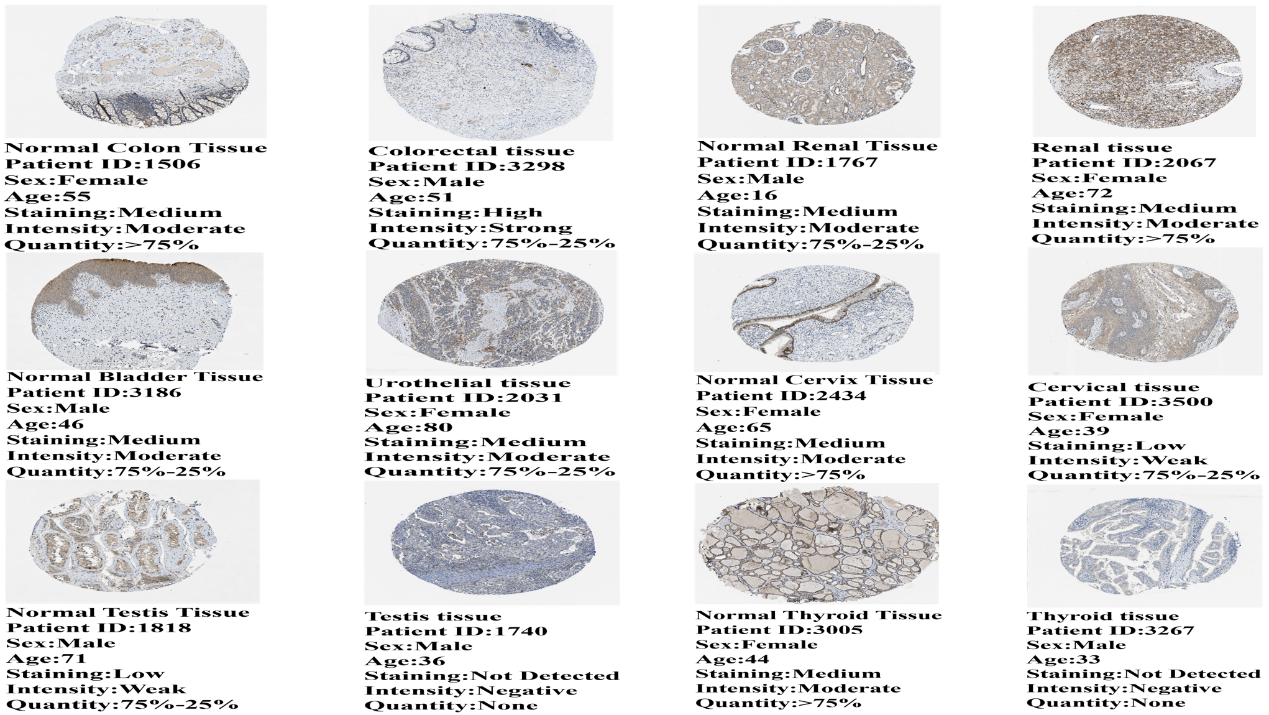


**Figure S2-C.** IHC images of normal and tumour tissues of RIPK2 from patients with Head and Neck cancer, Lung cancer, Liver cancer and Stomach cancer. All IHC images and patient information were derived from the HPA.


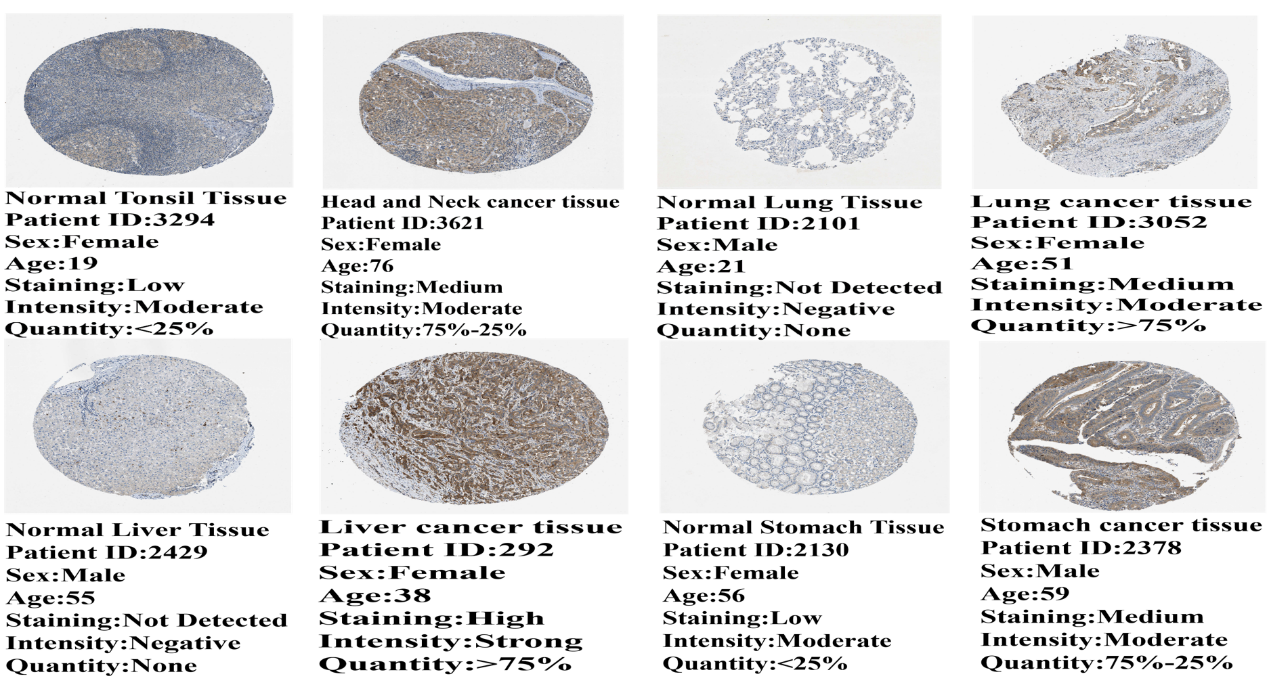


**Figure S3.** The disease-free survival of RIPK2 expression in KICH(p=0.044), KIRC(0.013), KIRP(p=0.0069), LUSC(p=0.044), SARC(p=0.019) and UVM(p=0.028). We obtained a relationship between RIPK2 and survival prognosis of cancer from GEPIA2. Kaplan-Meier curves were all positive. (TCGA dataset).


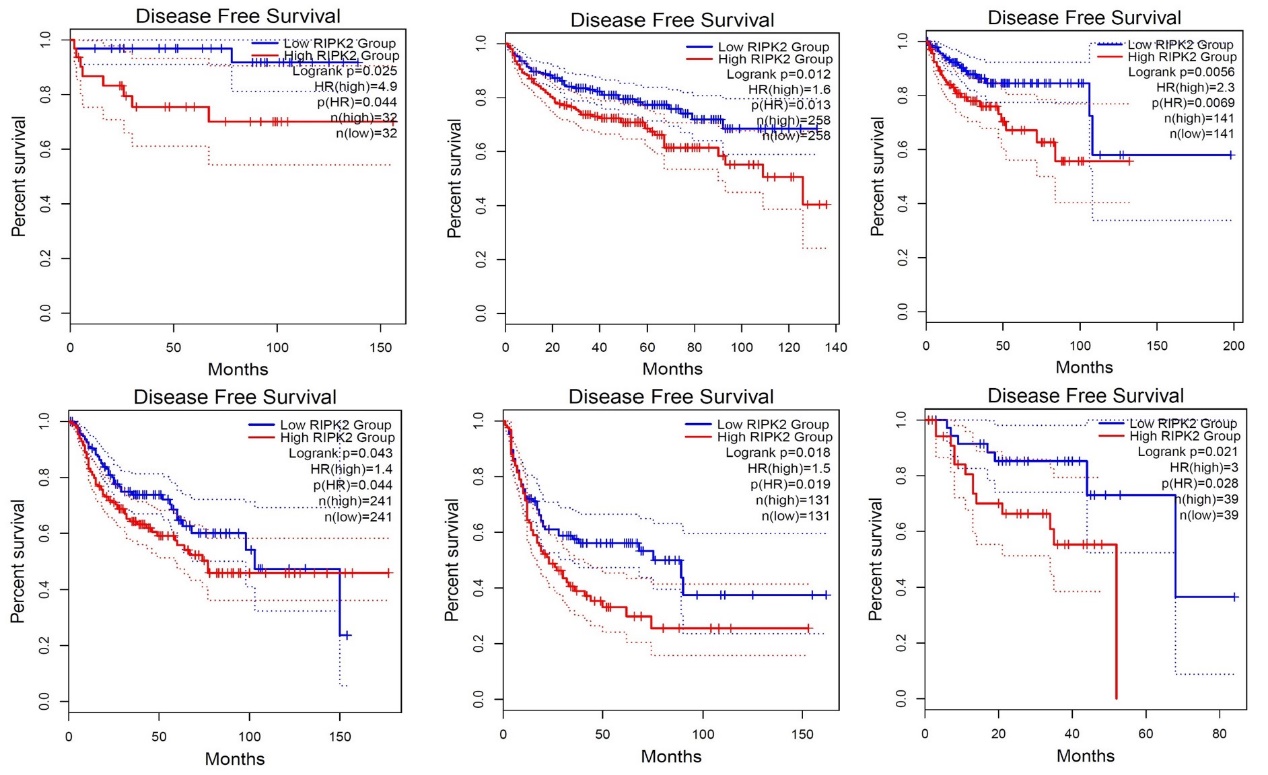


**Figure S4-A.** Correlation between RIPK2 mutation status and OV overall survival(P=1.691e-4), progression-free survival(P=0.0139), disease-free survival(P=0.0227) and disease specificity(P=2.379e-4). The solid yellow line represents RIPK2 altered group in tumour tissues, and the dash-dotted blue line represents RIPK2 unaltered group in tumour tissues. (TCGA dataset).


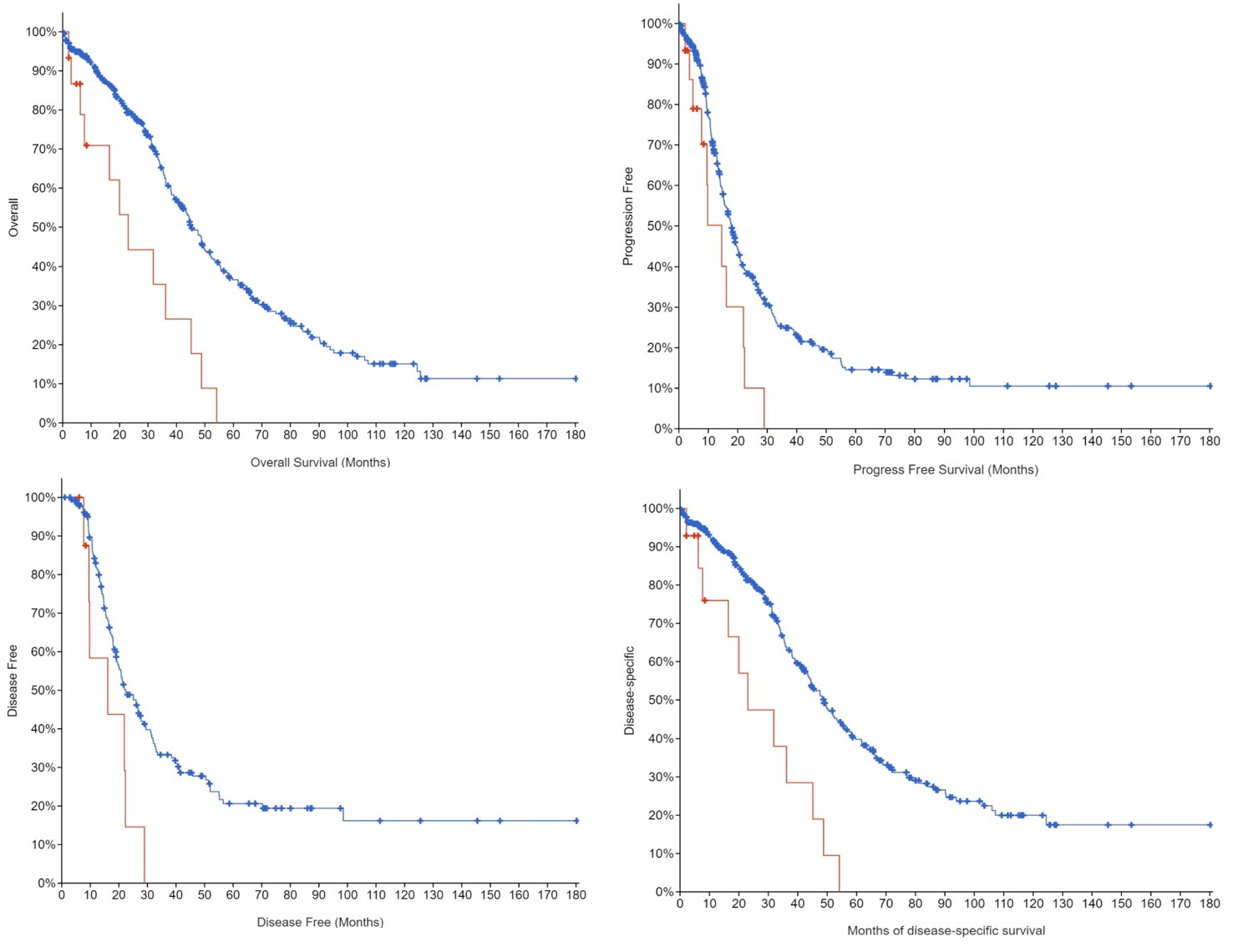


**Figure S4-B.** Correlation between RIPK2 mutation status and overall survival, progression-free survival, disease-free survival, and disease specificity of PAAD(OS:p=1.672e-3;PFS:p=5.91e-3;DF:p=2.96e-8;DSS:p=0.038) and PRAD(OS:p=0.0463;PFS:p=7.064e-4;DF:p=2.427e-5;DSS:p=1.881e-3). The solid yellow line represents RIPK2 altered group in tumour tissues, and the dash-dotted blue line represents RIPK2 unaltered group in tumour tissues. (TCGA dataset).


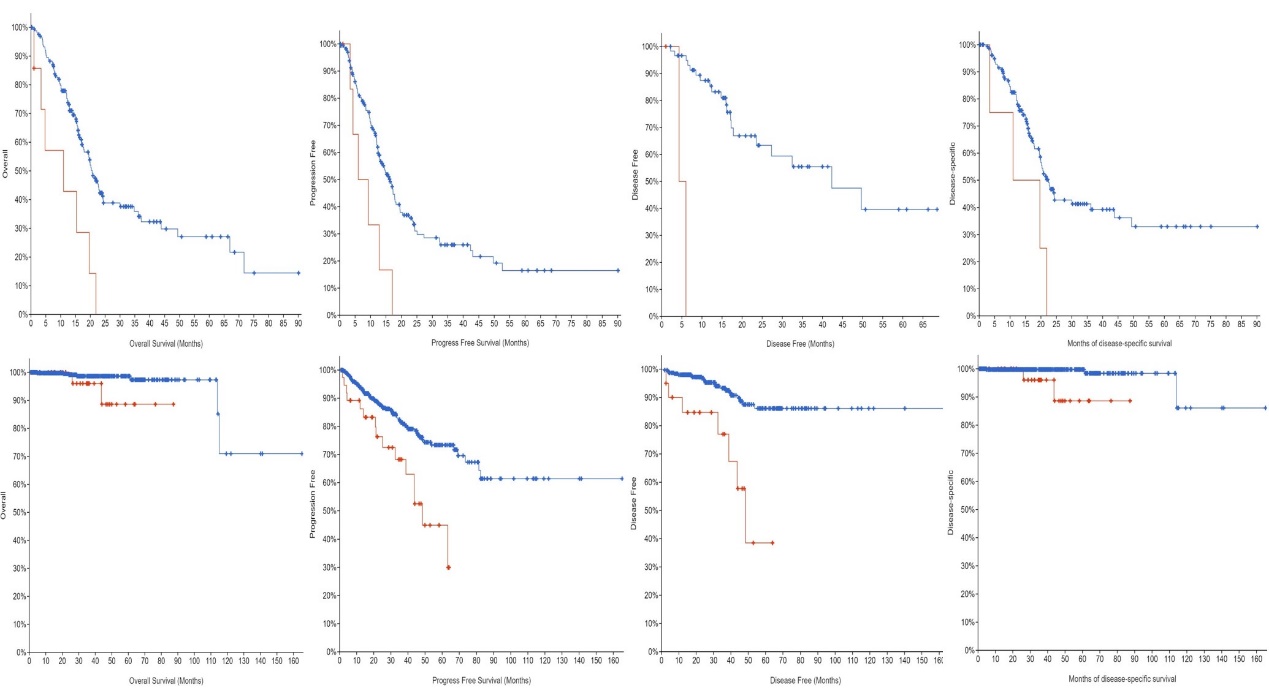


**Figure S5-A.** Correlation between the expression of RIPK2 and immune infiltration of Tregs cells. In LIHC,LUSC,TGCT, and THCA, RIPK2 expression was positively correlated with Tregs cells.（TCGA dataset）


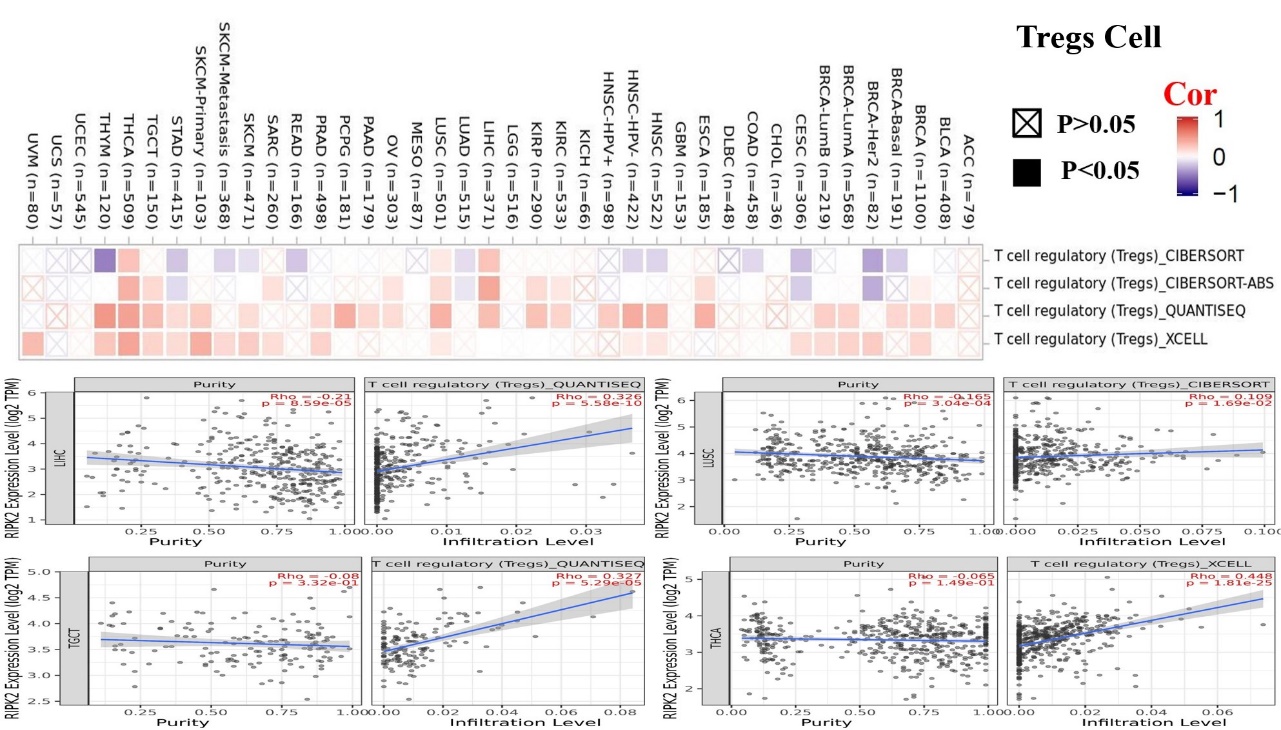


**Figure S5-B.** Correlation between expression of RIPK2 and immune infiltration in cancer-associated fibroblasts. In COAD, KIRP, and LUSC, RIPK2 expression was positively correlated with cancer-associated fibroblasts expression; However, in BRCA, RIPK2 expression was negatively correlated with cancer-associated fibroblasts cells.（TCGAdataset）.


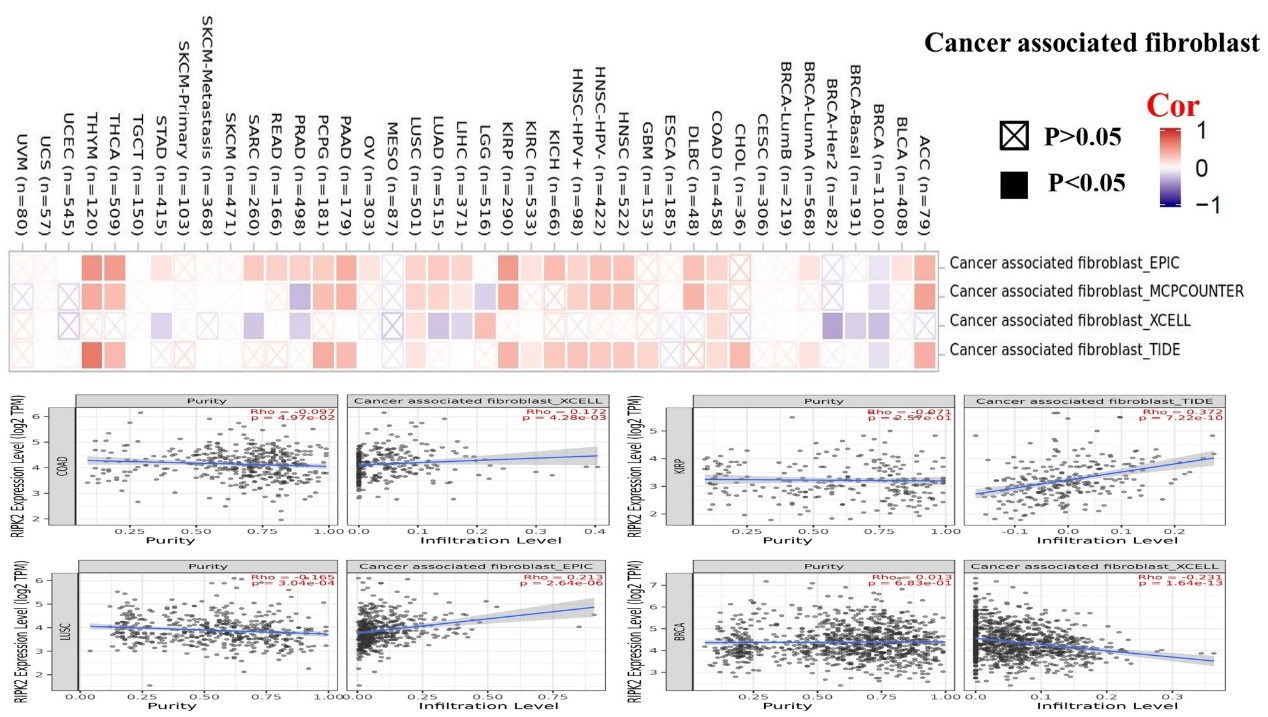


**Figure S5-C.** RIPK2 expression was positively correlated with GZMB, NGK7 and PRF1 expression in most cancers, but negatively correlated with PRAD and LGG, and had no correlation with SKCM and DLBC. （TCGAdataset）.


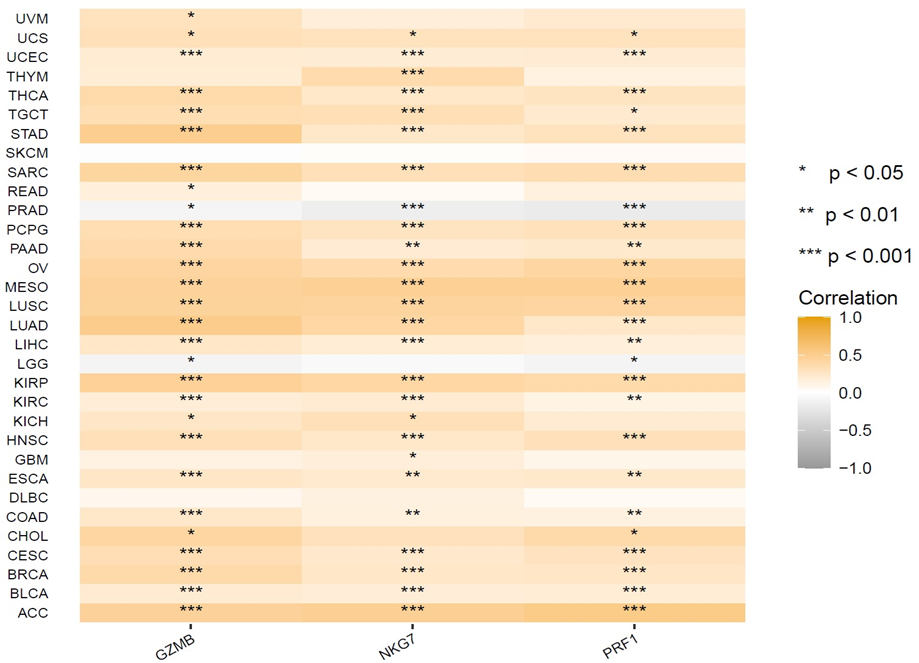


**Figure S6-A.** Correlation between the expression levels of RIPK2 and NBN, NUDCD1, DCAF13, GDI2 and OSGIN2 in all cancers. The results showed that the expression of RIPK2 was positively correlated with the expression of NBN, NUDCD1, DCAF13, GDI2 and OSGIN2.


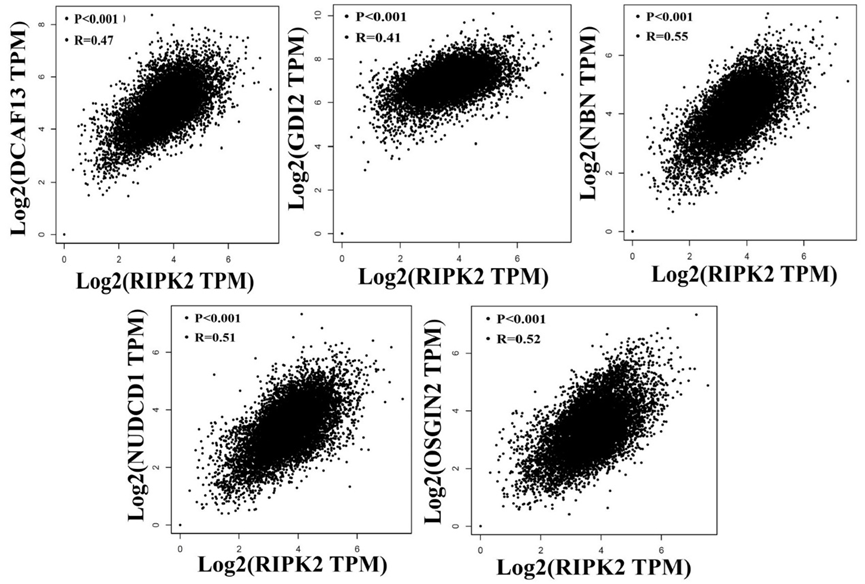


**Figure S6-B.** Heat map data between the expression levels of RIPK2 and DCAF13, GDI2, NBN, NUDCD1 and OSGIN2. The results showed that the expression of RIPK2 was positively correlated with the expression of DCAF13, GDI2, NBN, NUDCD1and OSGIN2.


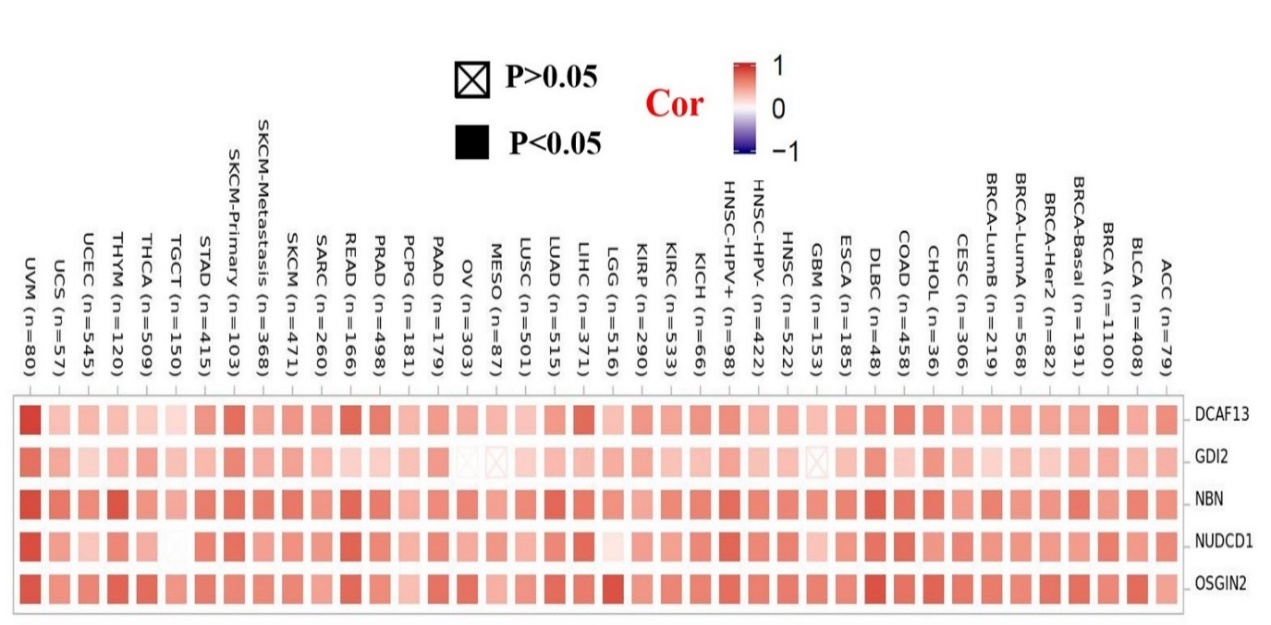


**Figure S6-C.** The GO (molecular functional MF) analysis results of RIPK2 interaction and expression related genes. The most important molecular functional are ubiquitin-like protein ligase binding and ubiquitin protein ligase binding.


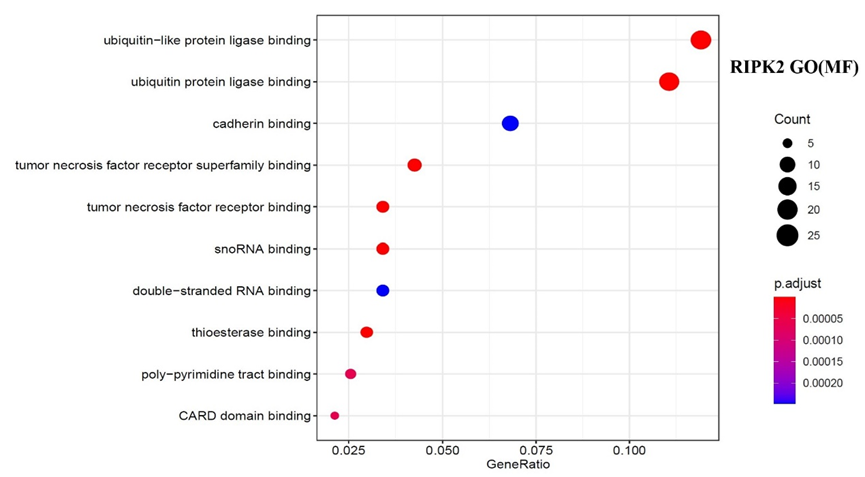


**Figure S6-D.** Go (Biological Process BP) analysis results of RIPK2 interaction binding and expression related genes. The most important biological process are I-kappaB kinase/NF-kappaB signaling and regulation of I-kappaB kinase/NF-kappaB signaling.


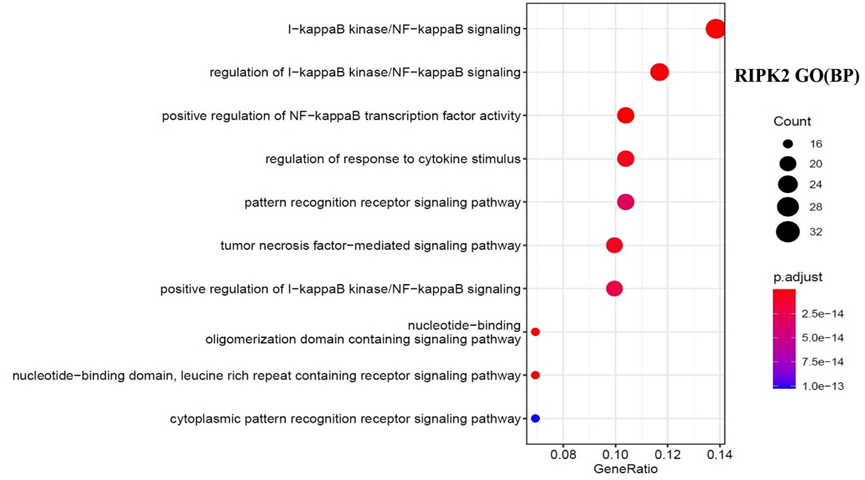


**Figure S6-E.** Go (Cellular Component CC) analysis results of RIPK2 interaction binding and expression related genes. The most important cellular component are ribonucleoprotein granule and cytoplasmic ribonucleoprotein granule.


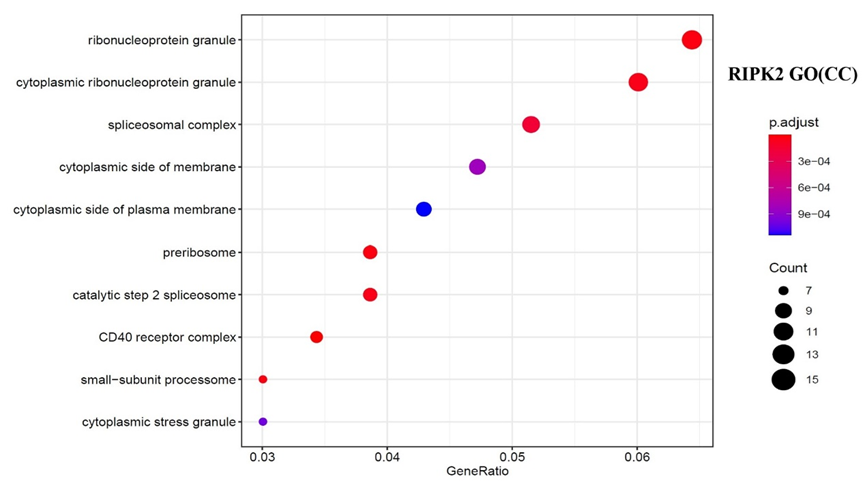

Supplement: Supplementary file 1 — Additional file 1. Supplementary figures. [file 12920_2022_1239_MOESM1_ESM.docx]
